# Genome-wide analysis of Smad and Schnurri transcription factors in *C. elegans* demonstrates widespread interaction and a function in collagen secretion

**DOI:** 10.1101/2024.06.05.597576

**Published:** 2024-06-06

**Authors:** Mehul Vora, Jonathan Dietz, Zachary Wing, Jun Liu, Christopher Rongo, Cathy Savage-Dunn

**Affiliations:** 1 Department of Biology, Queens College, CUNY, NY, USA; 2 PhD Program in Biology, the Graduate Center, CUNY, NY, USA; 3 Waksman Institute, Dept. of Genetics, Rutgers University, NJ, USA; 4 ModOmics Ltd, Southampton, UK; 5 Department of Molecular Biology and Genetics, Cornell University, NY, USA

## Abstract

Smads and their transcription factor partners mediate the transcriptional responses of target cells to secreted ligands of the Transforming Growth Factor-β (TGF-β) family, including those of the conserved bone morphogenetic protein (BMP) family, yet only a small number of direct target genes have been well characterized. In *C. elegans,* the BMP2/4 ortholog DBL-1 regulates multiple biological functions, including body size, via a canonical receptor-Smad signaling cascade. Here, we identify functional binding sites for SMA-3/Smad and its transcriptional partner SMA-9/Schnurri based on ChIP-seq peaks (identified by modEncode) and expression differences of nearby genes identified from RNA-seq analysis of corresponding mutants. We found that SMA-3 and SMA-9 have both overlapping and unique target genes. At a genome-wide scale, SMA-3/Smad acts as a transcriptional activator, whereas SMA-9/Schnurri direct targets include both activated and repressed genes. Mutations in *sma-9* partially suppress the small body size phenotype of *sma-3,* suggesting some level of antagonism between these factors and challenging the prevailing model for Schnurri function. A functional analysis of target genes revealed a novel role in body size for genes involved in one-carbon metabolism and in the endoplasmic reticulum (ER) secretory pathway, including the disulfide reductase *dpy-11.* Our findings indicate that Smads and SMA-9/Schnurri have previously unappreciated complex genetic and genomic regulatory interactions that in turn regulate the secretion of extracellular components like collagen into the cuticle to mediate body size regulation.

## INTRODUCTION

Members of the TGF-β family of secreted ligands play numerous roles in development and disease. In humans, there are 33 ligand genes that can be broadly separated into two subfamilies: the TGF-β/Activin subfamily and the BMP subfamily [1]. Due to the conservation of these ligands and their signaling pathways across metazoans, genetic studies in invertebrate systems have been instrumental in identifying signaling mechanisms [2, 3]. Canonical signaling occurs when ligand dimers bind to transmembrane receptors generating a heterotetrameric complex consisting of two type I and two type II serine/threonine kinase receptors. Following ligand binding and complex assembly, the constitutively active type II receptor phosphorylates the type I receptor on the GS domain and thereby activates its kinase domain [4]. The activated type I receptor phosphorylates the C-terminus of intracellular receptor-regulated Smads (R-Smads), promoting their heterotrimeric complex formation with co-Smads. The heterotrimeric Smad complex accumulates in the nucleus and binds DNA directly to elicit changes in gene expression [5–15]. Co-Smads for all ligands and R-Smads for TGF-β/Activin ligands bind a 4 bp GTCT Smad Binding Element (SBE); furthermore, R-Smads for BMP ligands associate with GC-rich sequences (GC-SBE) [16–18]. The SBE is considered too degenerate and low affinity to account fully for binding specificity, so transcription factor partners likely contribute to target gene selection [19]. To date, only a few direct target genes of Smads have been extensively studied, including Drosophila *brinker* [20]; Xenopus *mixer* [21] and *Xvent2* [22]; and the mammalian *ATF3* and *Id* genes [23]. Genome-wide studies have the potential to expand these examples and elucidate general principles of target gene selection [24–26]. More of these studies are needed to understand how Smad transcriptional partners influence target gene selection and contribute to the execution of specific biological functions.

In the nematode *Caenorhabditis elegans,* a BMP signaling cascade initiated by the ligand DBL-1 plays a major role in body size regulation [27]. In nematodes, body size is constrained by a collagen-rich cuticle, which is secreted by an epidermal layer (the hypodermis) and remodeled over four successive molts during larval growth and then continuously during adulthood [28, 29]. DBL-1 signals through type I receptor SMA-6, type II receptor DAF-4, and Smads SMA-2, SMA-3, and SMA-4 (founding members of the Smad family), which act together in the hypodermis to promote body size growth during the earliest larval growth stages [30–32]. The exact mechanism by which the DBL-1 pathway regulates body size is not fully understood, but is known to involve the regulated synthesis of cuticular collagen, of which there are over 170 genes [33–35]. A complete understanding of how DBL-1 regulates body size will require the identification of all direct transcriptional targets of the pathway during larval growth.

In addition to body size, the DBL-1 pathway also regulates male tail patterning, mesodermal lineage specification, innate immunity, and lipid metabolism. A transcription factor partner for this pathway, SMA-9, has been identified that plays a role in each of these biological functions [36, 37]. SMA-9 is the homolog of Drosophila Schnurri, which was identified for its roles in Dpp/BMP signaling [38–40]. Three vertebrate Schnurri homologs regulate immunity, adipogenesis, and skeletogenesis, acting through both BMP-dependent and BMP-independent mechanisms [41–44]. Schnurri proteins are very large transcription factors with multiple Zn-finger domains. At the *brinker* locus in Drosophila and the *Xvent2* locus in Xenopus, binding of an R-Smad and a Co-Smad with a precise 5 bp spacing between binding sites has been shown to recruit Schnurri, which controls the direction of transcriptional regulation [22]. This model for Smad-Schnurri interaction has not been tested at a genomic scale.

In this study, we use BETA software to combine RNA-seq and ChIP-seq datasets for SMA-3/Smad and SMA-9/Schnurri to identify direct versus indirect target genes of these factors, as well as to identify common versus unique targets [45, 46]. Analysis of *sma-3; sma-9* double mutants further extends our understanding of how these factors interact to produce locus-specific effects on target genes. We use GO term analysis and loss-of-function studies that shed light on the downstream effectors for body size regulation, lipid metabolism, and innate immunity. Finally, we use a ROL-6::wrmScarlet reporter for collagen synthesis and secretion to show that SMA-3, SMA-9, and the transcriptional target gene DPY-11 regulate body size growth by promoting the secretion and delivery of collagen into the cuticle.

## METHODS

### *C. elegans* strains

*C. elegans* strains were grown at 20°C using standard methods unless otherwise indicated. N2 is the wild-type strain; some strains were provided by the *Caenorhabditis Genetics Center* (CGC), which is funded by NIH Office of Research Infrastructure Programs (P40 OD010440) or generated in previous work. Strong loss-of-function or null alleles were used. The following genotypes were used: *sma-3(wk30), sma-9(ok1628), qcIs6[sma-3p::gfp::sma-3, rol-6(d)], jjIs1253[sma-9p::sma-9C2::gfp+unc-119(+)] (*generated via bombardment), [47]*, rrf-3(pk1426), fat-6(tm331), fat-7(wa36), nhr-114(gk849), acs-22(gk373989), lbp-8(gk5151), clec-60(tm2319), sysm-1(ok3236), ins-7(ok1573), sma-2(e502), rol-6(syb2235[rol-6::wrmScarlet])* [48]*, and pwSi82[hyp-7p::VIT2ss::oxGFP::KDEL, HygR].* The double mutants *sma-3(wk30); sma-9(wk97), and rrf-3(pk1426); lon-2(e678)* were generated in this study.

### RNA-seq

Developmentally synchronized animals were obtained by hypochlorite treatment of gravid adults to isolate embryos. Animals were grown on NGM plates at 20°C until the late L2 stage. Total RNA was isolated from animals using Trizol (Invitrogen) combined with Bead Beater lysis in three biological replicates for each genotype [45]. Libraries were generated using polyA selection in a paired-end fashion and sequenced on an Illumina HiSeq (2x150 bp configuration, single index, per lane) by Azenta (formerly Genewiz). Reads were mapped to the *C. elegans* genome (WS245) and gene counts generated with STAR 2.5.1a. Normalization and statistical analysis on gene counts were performed with EdgeR using generalized linear model functionality and tagwise dispersion estimates. Principal Component Analysis showed tight clustering within four biological replicates, with a clear separation between genotypes in which SMA-3 and SMA-9 active versus inactive. Likelihood ratio tests were conducted in a pairwise fashion between genotypes with a Benjamini and Hochberg correction. All RNA-seq raw sequence files as well as normalized counts after EdgeR can be accessed at GEO (Accession Number: GSE266398)

### Chromatin Immunoprecipitation Sequencing

Chromatin immunoprecipitation sequencing (ChIP-seq) was performed by Michelle Kudron (Valerie Reinke Model Organism ENCyclopedia Of DNA Elements and model organism Encyclopedia of Regulatory Networks group) on the *sma-3(wk30);qcIs6[GFP::SMA-3]* and LW1253*: jjIs1253[sma-9p::sma-9C2::gfp+unc-119(+)]* strains at the late L2 stage [49]. Data are available at encodeproject.org. To calculate distances between SMA-3 and SMA-9 ChIP-seq peaks, each peak was reduced to a centroid position (midpoint between the two border coordinates along the chromosome). For each chromosome, a matrix of SMA-3 and SMA-9 peak centroids was created, allowing the measurement of distance (in bps) between every SMA-3 and SMA-9 centroid along that chromosome. The shortest distance in the matrix was chosen to define each SMA-3/SMA-9 nearest neighboring pair. The resulting inter-centroid distances were analyzed from all 6 chromosomes. To mimic a random distribution of SMA-3 and SMA-9 peaks, each peak on a given chromosome was reassigned a location on that chromosome using randomized values (generated by the Microsoft Excel randomization function) within the size range for that chromosome. A matrix of SMA-3 and SMA-9 peak centroids was then analyzed from this randomized dataset as described above for the actual dataset.

### Identification of Direct Targets Using BETA and LOA

To identify SMA-3 direct targets, differentially expressed genes (DEGs) from the RNA-seq comparison of wild type vs. *sma-3(wk30)* using an FDR≤0.05 were compared against the genomic coordinates of SMA-3 peaks from the ChIP-seq analysis using BETA basic and the WS245 annotation of the *C. elegans* genome [46]. The following parameters were used: 3 kb from TSS, FDR cutoff of 0.05 and one-tail KS test cutoff of 0.05. The input files consisted of .bed files of IDR thresholded peaks and differential expression Log_2_FC and FDR values from the RNA-seq. An identical approach was used to identify SMA-9 direct targets using DEGs from the RNA-seq comparison of wild type vs. *sma-9(ok1628)*.

To identify direct targets co-regulated by both SMA-3 and SMA-9, the two pairwise RNA-seq comparisons (wild type vs. *sma-3* and wild type vs. *sma-9*) were analyzed, measuring DEGs for the same genes in both comparisons. Taking a conditional approach, the information from the first comparison (wild type vs. *sma-3*) was examined to see if it affected interpretation in the second (wild type vs. *sma-9*). Using the approach of Luperchio et al. [50], the genes in the second comparison were split into two groups, conditional on the results in the first comparison, with one group comprising genes found to show differential expression in the first comparison, and the second group comprising genes found not to show differential expression. To estimate which genes were differentially regulated, an FDR of 0.01 was used to generate an overlapping list between the two comparisons. BETA basic was then used to identify potential direct targets of the SMA-3/SMA-9 combination using just the ChIP-seq peaks that overlapped between the two transcription factors. The following parameters were used: 3 kb from TSS, FDR cutoff of 0.05 and one-tail KS test cutoff of 0.05. Analysis tools can be obtained at GitHub: https://github.com/shahlab/hypoxia-multiomics as per [45]. The WormBase database was used to obtain information about candidate target genes, including sequence, genetic map position, expression pattern, and available mutant alleles [51].

### Quantitative RT-PCR analysis

Worms were synchronized using overnight egg lay followed by 4-h synchronization. When animals reached L4 stage, they were collected and washed, and then RNA was extracted using previously published protocol [52] followed by Qiagen RNeasy miniprep kit (Catalogue. No. 74104). Invitrogen SuperScript IV VILO Master Mix (Catalogue. No.11756050) was used to generate cDNA, and qRT-PCR analysis was done using Applied Biosystems *Power* SYBR Green PCR Master Mix (Catalogue. No. 4367659). Delta delta Ct analysis was done using Applied Biosystems and StepOne software. All qRT-PCR analysis was repeated on separate biological replicates. The following primer pairs were used: 5’-ATGTGTGACGACGAGGTTGCC-3’ and 5’-GTCTCCGACGTACGAGTCCTT-3’ to detect *act-1*, 5’-GTGGATTCTTCTTCGCTCAT-3’ and 5’-CACAAGATGACAAGTGGGAA-3’ to detect *fat-6*, 5’-CATTCGATGTTTTTGAGGCG-3’ and 5’-GATCGAAGTAGGCACCATCT-3’ to detect *nhr-114*, and 5’-GGCAGGTCTAATCCACGACTTG-3’ and 5’-CTAATGTCCGGGTTCCCATCG-3’ to detect *C54E4.5*. All graphs were made using GraphPad Prism software and statistical analysis was performed using One-way ANOVA with Multiple Comparison Test, as calculated using the GraphPad software.

### RNAi Analysis of Body Size

RNAi knockdown of individual target genes was performed in the RNAi-sensitive *C. elegans* mutants *rrf-3(pk1426)* and *rrf-3(pk1426); lon-2(e678),* which were fed HT115 bacteria containing dsRNA expression plasmid L4440, with or without gene targeting sequences between flanking T7 promoters. NGM growth plates were used containing ampicillin for L4440 RNAi plasmid selection and IPTG (isopropyl β-D-1-thiogalactopyranoside) to induce dsRNA expression. Both *rrf-3(pk1426)* and *rrf-3(pk1426); lon-2(e678)* were exposed to the RNAi food during the L4 stage and allowed to lay eggs for 3 hours and then removed. Following hatching and development to adulthood exposed to the RNAi food, 2 adult hermaphrodites were transferred to fresh RNAi plates, allowed to lay eggs, and removed from the plate. Upon hatching and development to the L4 stage, hermaphrodites were imaged using an AxioImager M1m (Carl Zeiss, Thornwood, NY) with a 5X (NA 0.15) objective. The RNAi feeding constructs were obtained from the Open Biosystems library (Invitrogen). To analyze the body length of the RNAi exposed animals, 3 independent measurements were made per worm using the segmented line tool on Fiji/ImageJ [53]. 3–5 biological replicates were completed for each RNAi construct. The data were analyzed using ANOVA with Dunnett’s post hoc test correction for multiple comparisons.

### Hypodermal Imaging of ROL-6::wrmScarlet

Animals expressing *rol-6::wrmScarlet* in different genetic backgrounds were imaged using a Chroma/89 North CrestOptics X-Light V2 spinning disk, a Chroma/89North Laser Diode Illuminator, and a Photometrics PRIME95BRM16C CMOS camera via MetaMorph software. Day 1 adults (unless otherwise noted) were used to ensure molting was completed. A 63X oil objective (NA 1.4) was used to detect fluorescence. In order to visualize the cuticle and hypodermis layers of each animal, a z-series was completed using a 0.5 micron step size across 6.5 microns. Each image was analyzed using Fiji/ImageJ for fluorescence quantification in the hypodermis of the animals. Background was subtracted using a rolling ball filter. An outline was drawn around each nematode and the mean fluorescence intensity was calculated within the outline. At least 10 animals were analyzed and pooled across 3–4 biological replicates. Using GraphPad Prism, the individual mean fluorescence intensity values were normalized to the mean for control animals in each experiment and analyzed using ANOVA with Dunnett’s post hoc test correction for multiple comparisons. Images were then deconvolved using DeconvolutionLab2 [54].

Animals expressing *rol-6::wrmScarlet* together with the ER marker *VIT2ss::oxGFP::KDEL* were imaged using a Zeiss Elyra 7 Lattice SIM. A 60X water objective (NA 1.2) was used to detect fluorescence, and a z-series was completed as described above.

RNAi treatment of *rol-6::wrmScarlet* animals was performed similarly to the RNAi treatment in the body length analysis with these exceptions: nematodes exposed to *dpy-11* RNAi grew for one generation until day 1 adulthood before imaging, nematodes exposed to C54H2.5 and F41C3.4-containing RNAi plasmids were introduced to the animals at the L1 development stage and allowed to develop until day 1 adulthood, and nematodes exposed to Y25C1A.5 and Y113G7A.3-containing RNAi plasmids were introduced to animals at the L4 development stage and grown for 24 hours before imaging. Tunicamycin treatment of *rol-6::wrmScarlet* animals was completed by allowing animals to develop from eggs to L4 stage in the presence of 5 µg/mL in NGM plates. Experiments were conducted over 3–4 biological replicates. The data were analyzed using an unpaired two-tailed t test ANOVA with Dunnett’s post hoc test correction for multiple comparisons, where appropriate.

## RESULTS

### Transcription factors SMA-3 and SMA-9 bind overlapping and distinct genomic sites

Genetic evidence identifies SMA-3/Smad as a core component of the DBL-1/BMP signaling cascade [2]. For many identified functions, *sma-3* mutant phenotypes are indistinguishable from those of *dbl-1* ligand mutants, including body size, male tail patterning, mesodermal lineage specification, and fat storage [2, 36, 55]. In contrast, SMA-9 contributes to each of these functions, but in each case has a different effect, either a more limited role, a weaker effect, or an antagonistic phenotype [36, 37, 55]. We sought to determine the transcriptional targets of SMA-3 and SMA-9 on a genome-wide scale by analyzing ChIP-seq data from genome binding of GFP-tagged SMA-3 and SMA-9 expressed from transgenes [56]. These constructs are functional, as evidenced by their ability to rescue the mutant phenotypes of respective loss-of-function *sma-3* and *sma-9* mutants [36, 55]. Genome binding was analyzed at the second larval (L2) stage, a developmental stage at which a Smad reporter is highly active, and both SMA-3 and SMA-9 are first observed to affect body size [57]. ChIP sequencing reads identify 4205 peaks for GFP::SMA-3 and 7065 peaks for SMA-9::GFP (Supplementary File 1).

**Figure 1.**
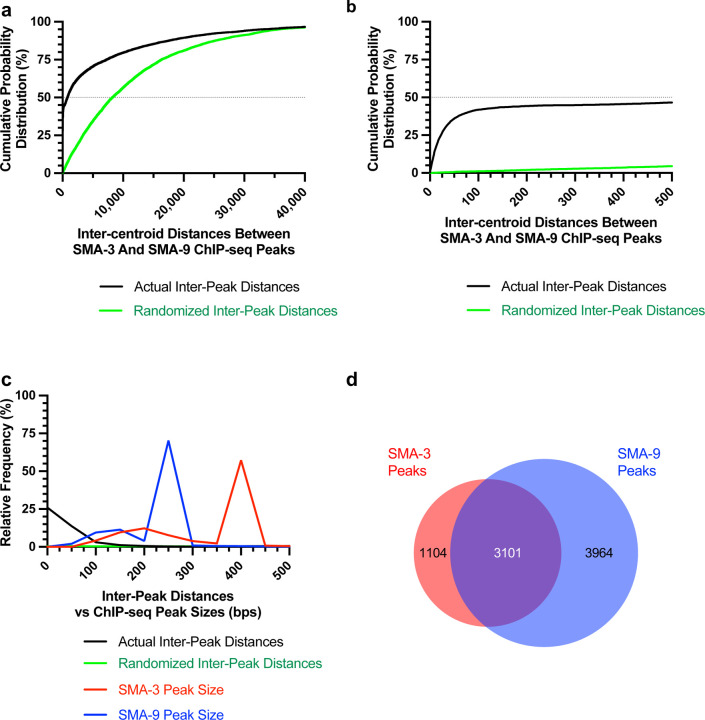
Transcription factors SMA-3 and SMA-9 bind both overlapping and distinct genomic sites (a) Cumulative probability distribution graph of the distances between the centroids (base pair position located centrally within each peak) of nearest neighbor SMA-3 and SMA-9 ChIP-seq peaks. The black line represents actual inter-peak distances, whereas the green line represents a hypothetical randomized dataset. The horizontal dotted line indicates the point in the curve at which half of the peak pairs fall. (b) Same cumulative probability distribution as in (a), but focused on distances less than 500 bps. The centroids of nearly half of all SMA-3/SMA-9 neighboring pairs fall within 500 bps of each other. (c) Histogram of the interpeak distances (actual data in black, randomized data in green), as well as the ChIP-seq peak widths (SMA-3 in red, SMA-9 in blue). Most peaks are larger in size than most interpeak centroid distances, indicating substantial peak overlap. (d) Size proportional Venn diagram showing the number of SMA-3 and SMA-9 peaks that either overlap with one another or remain independent. Although most SMA-3 peaks overlap with SMA-9 peaks, more than half of SMA-9 peaks are located independent from SMA-3 peaks.

We considered the possibility that SMA-3 and SMA-9 bind as a protein complex in some contexts. In *Drosophila*, the SMA-9 homolog Schnurri interacts physically with the Smads Mad and Medea [58, 59]. We therefore analyzed the distances between the centroids of SMA-3 and SMA-9 ChIP-seq peaks. If SMA-3 and SMA-9 bind independently, then we would expect a Gaussian distribution of inter-centroid distances, whereas if they bind in a complex, we should see a non-Gaussian distribution with increased representation of distances less than or equal to the average peak size. This analysis demonstrated an increased representation (approaching 45%) of inter-centroid distances of 100 bp or less (Figure 1a,b), smaller than the average peak size for SMA-3 (400 bp) or SMA-9 (250 bp) (Figure 1c), consistent with the interpretation that SMA-3 and SMA-9 frequently bind as subunits in a complex. The midpoint of the cumulative probability distribution of inter-centroid distances was 788 bp; by contrast, a randomization of the positions of SMA-3 and SMA-9 ChIP peaks expanded the midpoint of the cumulative probability distribution of inter-centroid distances to 8,211 bp (Figure 1a,b). From this analysis, a substantial subset (3101 peaks) of the SMA-3 (73.7%) and SMA-9 (43.9%) peaks overlap (Figure 1d). We therefore considered these instances of overlapping peaks to be complexes, whereas adjacent but non-overlapping peaks likely represent independent binding, leading us to conclude that (1) SMA-3 typically binds to sites in complex with SMA-9, and that (2) over half of all SMA-9 sites are independent from these SMA-3/SMA-9 complex sites.

### Identification of direct transcriptional targets of SMA-3 and SMA-9

**Figure 2.**
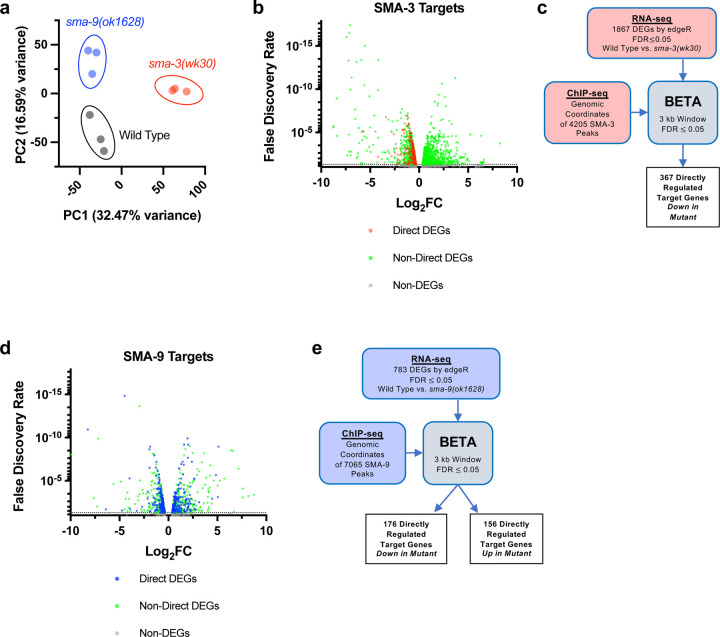
Identification of direct transcriptional targets of SMA-3 and SMA-9 (a) Principal Component Analysis (PCA) over two dimensions (PC1 and PC2) for RNA-seq datasets for wild type (in black), *sma-3(wk30)* (in red), and *sma-9(ok1628)* (in blue). The percent of variance for each component is indicated. The three biological replicates for each genotype are well clustered. (b) Volcano plot of RNA-seq FDR values versus log2 fold change (FC) expression for individual genes (squares) in *sma-3* mutants relative to wild type. The direct targets identified by BETA are indicated with red squares; the negative log2 FC values demonstrate that SMA-3 promotes the expression of these genes. Non-target genes nevertheless showing differential expression are indicated with green squares. (c) Strategy for integrating SMA-3 ChIP-seq and mutant RNA-seq data to identify directly regulated targets. (d) Volcano plot of RNA-seq FDR values versus log2 fold change expression for individual genes (squares) in *sma-9* mutants relative to wild type. The direct targets identified by BETA are indicated with blue squares; the combination of positive and negative log2FC values demonstrates that SMA-9 promotes the expression of some of these genes and inhibits the expression of others. Non-target genes nevertheless showing differential expression are indicated with green squares. (e) Strategy for integrating SMA-9 ChIP-seq and mutant RNA-seq data to identify directly regulated targets.

To determine how these binding sites correlate with changes in gene expression of neighboring genes, we performed RNA-seq on L2 stage samples of *sma-3* and *sma-9* mutants compared with wild-type controls. Principal component analysis (PCA) demonstrated that all three biologically independent replicates of each genotype clustered together (Figure 2a) and that each genotype is transcriptionally distinct from the others. Using a false discovery rate (FDR) ≤ 0.05, we identified 1093 differentially expressed genes (DEGs) downregulated and 774 upregulated DEGs in *sma-3* mutants (Figure 2b, Supplementary File 2). In *sma-9* mutants, we identified 412 downregulated DEGs and 371 upregulated DEGs (Figure 2d, Supplementary File 2).

RNA-seq identifies both direct and indirect transcriptional targets. To identify direct functional targets of each of these transcription factors, we employed BETA software (Figure 2c,e), which infers direct target genes by integrating ChIP-seq and RNA-seq data [46]. BETA analysis identified 367 direct targets for SMA-3 and 332 direct targets for SMA-9 (Supplementary File 3). Every identified direct target of SMA-3 was downregulated in the *sma-3* mutant (Figure 2b), indicating that SMA-3/Smad functions primarily as a transcriptional activator. In contrast, 46% of direct targets of SMA-9 were upregulated and 53% were downregulated in the *sma-9* mutant (Figure 2d, Supplementary File 3). Thus, SMA-9 likely acts as either a transcriptional activator or repressor depending on the genomic context. This conclusion is consistent with our previous analyses of SMA-9 function *in vivo* and in a heterologous system [34].

### Significant overlap in directly regulated DEGs of SMA-3 and SMA-9

**Figure 3.**
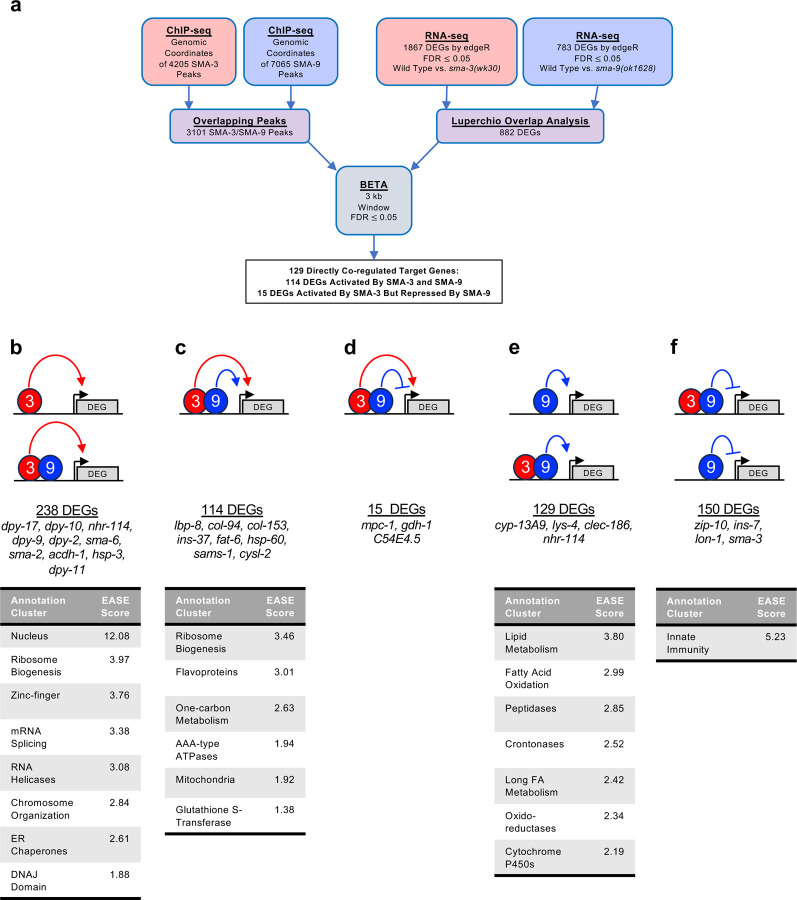
Significant overlap in directly regulated DEGs of SMA-3 and SMA-9 (a) Strategy for integrating SMA-3 and SMA-9 ChIP-seq and mutant RNA-seq data to identify common versus unique directly regulated targets. (b-f) Cartoon representations of the different types of direct target genes, their neighboring SMA-3 and/or SMA-9 binding sites, and the effect of those sites on that gene’s expression. The red circle labeled “3” represents SMA-3 binding sites, whereas the blue circle labeled “9” represents SMA-9 binding sites. Arrows represent that the wild type transcription factor promotes the expression of the neighboring DEG (gray), whereas T-bars indicate that it inhibits the expression of the DEG. Types of regulation include (b) SMA-3 alone promoting DEG expression, (c) SMA-3 and SMA-9 combined promoting expression, (d) SMA-3 and SMA-9 showing antagonistic regulation of expression, (e) SMA-9 alone promoting DEG expression, and (f) SMA-9 alone inhibiting DEG expression. Example DEGs and tables of annotation clusters for gene ontology terms for those DEGs (via DAVID, with accompanying statistical EASE score) are shown under the cartoon demonstrating each type of regulation.

Because analysis of ChIP-seq peaks suggests that SMA-3 and SMA-9 can bind DNA as a complex, we sought to identify a core subset of DEGs co-regulated by these transcription factors. Rather than relying on individual RNA-seq analyses, in which arbitrary cut-offs for significance may lead to an underestimation of the overlap, we performed Luperchio Overlap Analysis (LOA) on *sma-3* and *sma-9* RNA-seq datasets to identify 882 shared DEGs (Supplementary File 4) [45, 50]. From ChIP-seq data, we identified 3101 peaks that are overlapping between SMA-3 and SMA-9. We used LOA to identify DEGs shared between the pairwise comparison of *sma-3* vs wild type, and between the pairwise comparison of *sma-9* vs wild type, using evidence of potential DEGs in one comparison to inform the state of those potential DEGs in the other comparison. Processing common occupancy sites with the common DEGs through BETA software (Figure 3a), we identified 129 co-regulated direct target genes with overlapping SMA-3 and SMA-9 binding peaks (Supplementary File 5). These results are consistent with SMA-3 and SMA-9 acting broadly in a protein complex. Most (114) of these direct targets are activated by both SMA-3 and SMA-9 (Figure 3c), but 15 of them have reversed regulation in *sma-9* mutants compared with *sma-3* (Figure 3d), suggesting an antagonistic function that we analyze further below. For shared activated targets, loss of SMA-3 caused a greater fold change than loss of SMA-9 (Supplementary File 2).

Our BETA/LOA analysis next allowed us to deduce SMA-3 and SMA-9 exclusive targets, revealing 238 SMA-3-exclusive direct targets (Figure 3b) and 279 SMA-9-exclusive direct targets (Figure 3e,f). Surprisingly, many of these target genes contained overlapping SMA-3 and SMA-9 binding peaks (Figure 3b,e,f), although loss of one of the two factors did not result in changes in gene expression, perhaps suggesting that the presence of the other factor at these targets was sufficient to regulate gene expression to physiological levels. Interpretation is further complicated for target genes surrounded by a mixture of distinct and overlapping SMA-3 and SMA-9 peaks. Our results suggest that (1) SMA-3 and SMA-9 can act independent of one another, (2) a SMA-3/SMA-9 complex usually (although not always) acts as a transcriptional activator of shared target genes, and that (3) SMA-9 can act as either a transcriptional activator or repressor of its own SMA-3-independent targets.

### Mechanisms of integration of SMA-3 and SMA-9 function

**Figure 4.**
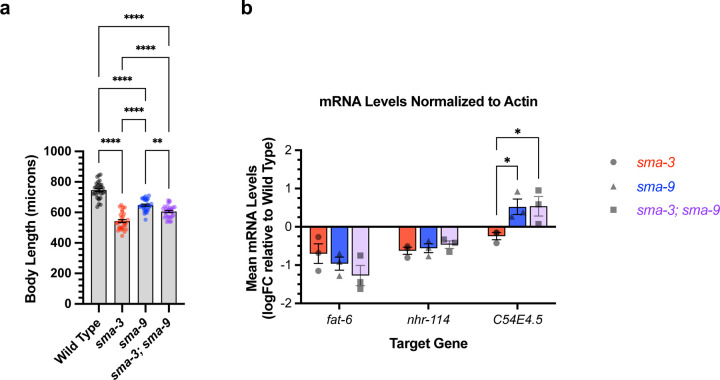
Genetic interactions between SMA-3 and SMA-9 (a) Mean body length of L4 animals (measured head to tail in microns). Dots indicate the size of individual animals. ****P<0.0001, **P<0.01 One way ANOVA with Tukey’s multiple comparison test. (b) Mean mRNA levels for the indicated target gene (X-axis) for the indicated genotypes (*sma-3*, *sma-9*, or the double mutant combination) relative to the level in wild type. Expression values are in log2 fold change (FC). Individual genotype mRNA levels for each gene were first normalized to actin mRNA levels in that genotype. *P<0.05 Two way ANOVA with Tukey’s multiple comparison test.

SMA-3 and SMA-9 both regulate body size. We used double mutant analysis to determine whether they do so independently or together. For two gene products that act together in the same pathway, we expect the double mutants to resemble one of the single mutants. If they function independently, then we expect the double mutant to be more severe than the single mutants (additive phenotypes). We constructed a *sma-3; sma-9* double mutant and measured its body length at the L4 stage in comparison with control, *sma-3* mutants, and *sma-9* mutants. Contrary to expectations, the double mutant was neither the same as nor more severe than the single mutants; instead, it showed an intermediate phenotype (Figure 4a). This result suggests that there may be some antagonistic action between these two transcription factors, consistent with our observation that some direct targets are regulated in opposite directions by SMA-3 and SMA-9.

We hypothesized that the interaction between SMA-3 and SMA-9 may be context dependent, with different target genes showing independent, coordinated, or antagonistic interactions between the transcription factors. We tested this hypothesis by performing qRT-PCR on select target genes in wild type, *sma-3* mutants, *sma-9* mutants, and *sma-3; sma-9* double mutants at the L2 stage. We considered three target genes: *fat-6*, which has overlapping SMA-3 and SMA-9 peaks and is downregulated in both *sma-3* and *sma-9* mutants; *nhr-114*, which has distinct non-overlapping SMA-3 and SMA-9 peaks and is downregulated in both *sma-3* and *sma-9* mutants; and C54E4.5, which has overlapping SMA-3 and SMA-9 peaks yet show opposite direction of regulation in *sma-3* versus *sma-9* mutants. For two of the tested target genes, *fat-6* and *nhr-114,* there was no significant difference in expression levels between the single and double mutants (Figure 4b), consistent with the transcription factors acting together, regardless of overlapping binding. The third target gene, C54E4.5, was selected because it is a co-direct target yet the RNA-seq data shows changes in its expression in opposite directions in *sma-3* versus *sma-9* mutants, downregulated in *sma-3* yet upregulated in *sma-9* relative to wild type. In the double mutant, C54E4.5 is upregulated and indistinguishable from the expression in *sma-9* single mutants (Figure 4b). Thus, for this target gene, the *sma-9* loss-of-function phenotype is epistatic to that of *sma-3.* This relationship is possible if SMA-9 is required for SMA-3 binding at this site, or if SMA-9 is required for SMA-3 to engage with the transcriptional machinery.

### Biological functions of SMA-3 and SMA-9 target genes

**Figure 5.**
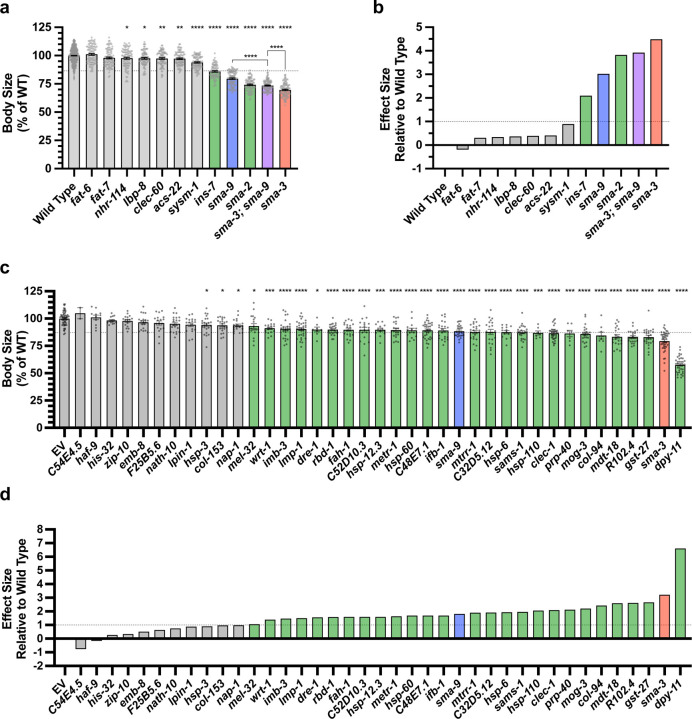
Multiple SMA-3 and SMA-9 target genes regulate body size (a) Mean body length of L4 animals (normalized to wild type) for the indicated mutants. Dots indicate the size of individual animals. Asterisks above each column indicate one way ANOVA with Dunnett’s multiple comparison test against wild type (****P<0.0001, ***P<0.001, **P<0.01, *P<0.05). Asterisks over pairwise comparison bars indicate one way ANOVA with Sídák’s multiple comparison test. The dotted line indicates the size value falling two standard deviations below the mean of wild type. (b) Glass’ effect size (the difference between the mean of the mutant and the mean for wild type, divided by the standard deviation of wild type) for the indicated mutants. The dotted line indicates an effect size of one. (c,d) Mean body size and Glass’ effect size for RNAi knockdowns of the indicated gene in the *rrf-3* RNAi sensitive strain. “EV” indicates the empty RNAi vector as a negative control. Asterisks above each column indicate one way ANOVA with Dunnett’s multiple comparison test against EV (****P<0.0001, ***P<0.001, **P<0.01, *P<0.05). For all graphs, red, blue, and purple columns indicate values for *sma-3* (mutant or RNAi knockdown), *sma-9* (mutant or RNAi knockdown), and their double mutant combination, respectively. Green bars indicate genotypes for which the mutant or RNAi knockdown resulted in a statistically significant reduction (P<0.05) with a Glass’ effect size of one or greater.

Gene Ontology analysis showed that SMA-3 and SMA-9 shared some annotation clusters, including for fatty acid metabolism and one-carbon metabolism, but also showed that each regulated its own annotation clusters, including collagen genes, ribosome biogenesis, mitochondrial proteins, and ER chaperones for SMA-3, as well as innate immunity factors and cytochrome P450s for SMA-9 (Figure 3b–f; Figure 3 – figure supplement 1). A Smad-independent role for SMA-9 in immunity is consistent with the pronounced role of vertebrate Schnurri homologs in immunity [42], which have not been reported to overlap with TGF-β-regulated functions. We expect that clusters of genes involved in fatty acid metabolism and innate immunity mediate the physiological functions of BMP signaling in fat storage and pathogen resistance, respectively. Using a candidate gene approach, we previously identified several cuticle collagen genes that mediate regulation of body size downstream of BMP signaling. Here, we sought to validate and extend this analysis in an unbiased manner by screening target genes for a function in body size regulation. For nine genes for which mutants were available, we measured body length at the L4 stage. As expected, *sma-2/Smad* mutants were smaller. As we previously showed, *fat-6* and *fat-7* mutants were not significantly different from wild type in larval stages [60]. Of the remaining six genes for which mutants were available, only *ins-7* showed significantly reduced body size with a statistical effect size (Glass’ effect size: the difference between the mean of the mutant and the mean for wild type, divided by the standard deviation of wild type) greater than one (Figure 5a,b). To test the functions of genes for which mutants were not available, we used RNAi depletion in the *rrf-3* RNAi hypersensitive mutant. If target genes truly act downstream of the DBL-1 signaling pathway to regulate body size, then we expect them to also act downstream of the negative regulator LON-2, which antagonizes the DBL-1 pathway with respect to body size at the level of ligand-receptor interactions. Thus, we also performed RNAi depletion in a *lon-2; rrf-3* double mutant, which demonstrates exaggerated DBL-1 signaling and elongated body size; we reasoned that candidate transcriptional effectors of the DBL-1 pathway might be more limiting and hence show a suppression phenotype in this genetic background. As expected, RNAi of *sma-3* or of *sma-9* reduced the body length in both genetic backgrounds (Figure 5b,c,d; Figure 5 – figure supplement 1). RNAi of one of the target genes, *mttr-1,* prevented the development of *rrf-3; lon-2* animals to the L4 stage, so body length could not be quantified at this stage. Nearly two thirds of the 36 genes tested caused a statistically significant reduction in body length (with a statistical effect size greater than one) upon RNAi treatment in at least one of the two genetic backgrounds. Half of the genes tested caused a significant reduction in body length in both genetic backgrounds (Figure 5 – figure supplement 1), making them strong candidates for direct transcriptional effectors of body size regulation. These genes have GO terms associated with either one-carbon metabolism or chaperone/ER secretion, suggesting that the upregulation of these activities is a key aspect of how DBL-1 signaling promotes growth.

### DBL-1 signaling promotes body size through collagen secretion

We observed that *dpy-11* depletion via RNAi resulted in the most severe reduction in body length among the tested target genes. The *dpy-11* gene encodes a protein-disulfide reductase involved in cuticle development. As we previously established that BMP signaling regulates cuticle collagen gene expression, we posited whether BMP signaling also impacts cuticle collagen secretion. We tested this hypothesis by monitoring the expression and localization of a cuticle collagen, choosing to monitor ROL-6, a cuticle collagen gene with a demonstrated role in body size [35].

**Figure 6.**
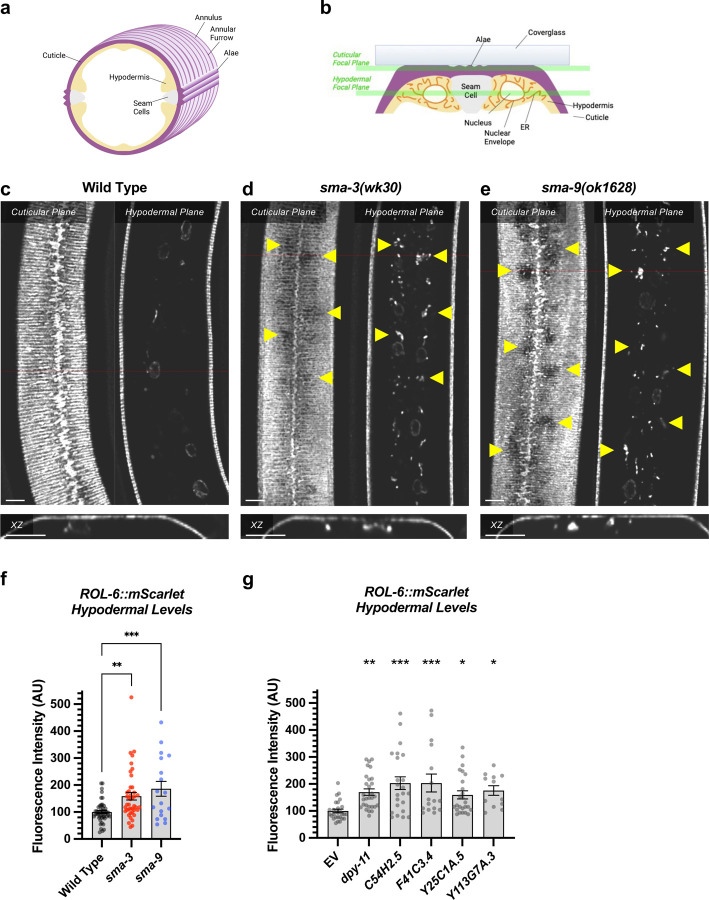
DBL-1 signaling promotes body size through collagen secretion (a) Cartoon illustrating a cross section through the nematode body (dorsal oriented up). Rings of cuticular collagen annuli (magenta), secreted by the underlying hypodermal cell layer (tan) surround the body. Lateral alae containing collagen, secreted by the underlying seam cells (gray), run orthogonal along the length of the body. (b) Cartoon illustrating a cross section through a portion of the nematode body (lateral oriented up) underneath a microscope coverglass. Horizontal green bars indicate the cuticular versus hypodermal plates captured by confocal microscopy in panels c-e. (c) ROL-6::wrmScarlet fluorescence detected in annuli and alae in the cuticular layer, as well as in nuclear envelopes in the underlying hypodermal layer in wild-type animals. The horizontal red line indicates the specific xz cross section shown below the cuticular and hypodermal plane panels. The bar indicates 5 microns. (d) ROL-6::wrmScarlet in *sma-3* mutants, visualized as per wild type. Patches of cuticular surface show diminished levels of ROL-6:wr:mScarlet, whereas the protein is detected in the hypodermal layer just under these patches (yellow arrowheads), suggesting a failure to deliver collagen to the surface cuticle (easily visualized in the xz panel). (e) Mutants for *sma-9* show the same phenotype as *sma-3*. (f) Quantification of ROL-6::wrmScarlet fluorescence in the hypodermal layer of indicated mutants. Dots indicate the fluorescence of individual animals. Asterisks over pairwise comparison bars indicate one way ANOVA with Sídák’s multiple comparison test (***P<0.001, **P<0.01). (g) A similar analysis for RNAi knockdowns of the SMA-3 target gene *dpy-11*, as well as four known ER secretion factors as comparative controls. Asterisks above each column indicate one way ANOVA with Dunnett’s multiple comparison test against wild type (***P<0.001, **P<0.01, *P<0.05).

**Figure 7.**
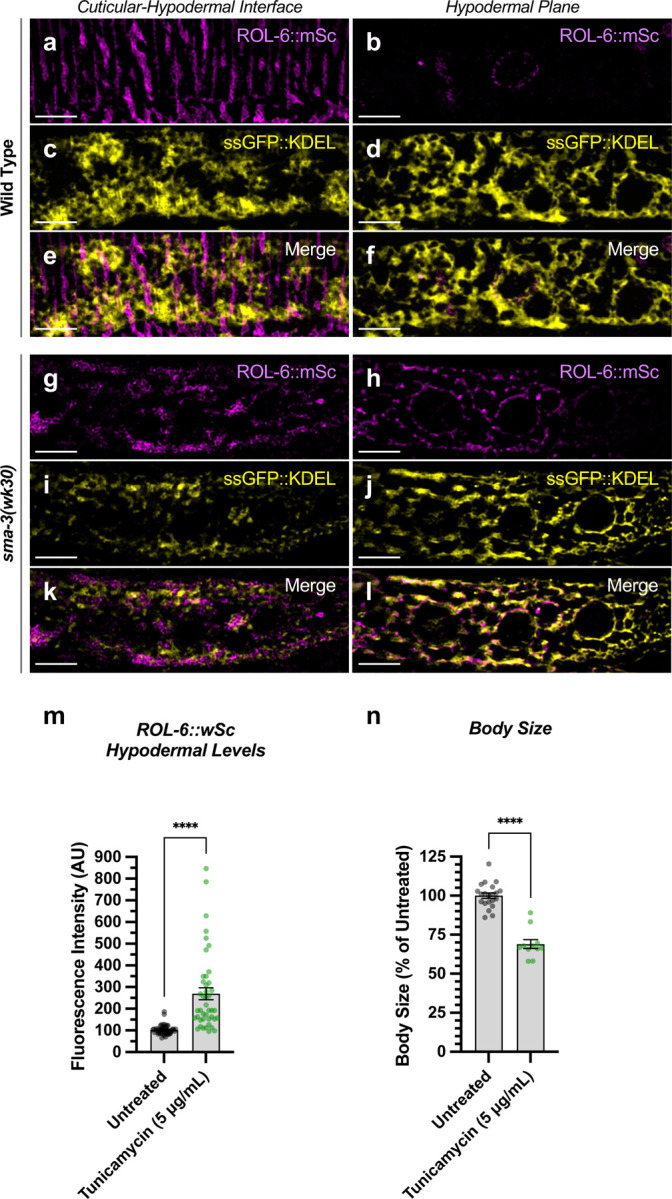
ROL-6::wrmScarlet accumulates in the ER of *sma-3* mutants ROL-6::wrmScarlet (magenta) and ssGFP::KDEL (an ER marker, shown here in yellow) shown separately (a-d, g-j) or merged (e-f, k-l) in either (a-f) wild type or (g-l) a *sma-3* mutant. (a,c,e,g,i,k) shows the interface between the cuticle and the hypodermis, as visualized by SIM super resolution microscopy. (b,d,f,h,j,l) shows the hypodermal layer at a focal plane centered around the nuclear envelope. In wild type, most ROL-6::wrmScarlet is delivered into the cuticle. In sma-3 mutants, lower levels of ROL-6::wrmScarlet are present in the cuticle and rapidly bleached under the SIM laser even under low power, whereas abundant ROL-6::wrmScarlet colocalized with the ER ssGFP::KDEL marker (yellow). We noted that ER reticulation in *sma-3* was thinner and skeletonized compared to wild type, perhaps suggesting reduced secretory throughput. (m) Quantification of ROL-6::wrmScarlet fluorescence in the hypodermal layer of tunicamycin treated versus untreated nematodes. (n) Mean body length of L4 animals (normalized to untreated) for tunicamycin treated versus untreated nematodes. (m,n) Dots indicate the values for individual animals. Asterisks over pairwise comparison bars indicate a student t test (****P<0.0001).

The hypodermis synthesizes and secretes collagen into the cuticle in a pattern of circular annuli that surround the animal along its length (Figure 6a). These collagen-rich annuli and the newly synthesized collagen inside the hypodermal cells underneath the cuticle can be distinguished using confocal microscopy (Figure 6b). We generated a functional endogenously tagged allele of *rol-6* that expresses a ROL-6::wrmScarlet fusion protein, taking care to preserve the proteolytic processing sites such that wrmScarlet remains attached to the final protein product. As previously shown [48], this fusion protein localizes to the cuticle (Figure 6c). In L4 animals, total cuticular fluorescence was reduced in *sma-3* and *sma-9* mutants, with clear patches of decreased ROL-6::wrmScarlet (Figure 6d,e). Interestingly, the hypodermal subcellular distribution of ROL-6::wrmScarlet was altered in *sma-3* and *sma-9* mutants, with ROL-6::wrmScarlet protein accumulating intracellularly in these mutants compared to wild type, often underneath the clear patches observed in the cuticular layer (Figure 6f). This phenomenon is consistent with changes in collagen secretion in impaired BMP signaling. We used structured illumination super-resolution microscopy to compare an endoplasmic reticulum (ER) marker, *KDEL::oxGFP* [61], with subcellular ROL-6::wrmScarlet, and we found that ROL-6 becomes trapped in and accumulates at the hypodermal ER in *sma-3* mutants (Figure 7a–l). We also noted that ER structures in *sma-3* mutants were thinner and more skeletonized relative to wild type (Figure 7j,l), consistent with a defect in secretion.

To further assess BMP signaling targets impact on collagen release, we depleted *dpy-11* via RNAi in nematodes expressing ROL-6::wrmScarlet. Consistent with a role for DPY-11 in the secretion of collagens, including ROL-6, we observed a severe disruption in both intracellular and cuticular deposition of ROL-6::wrmScarlet (Figure 6g; Figure 6 – figure supplement 1). To validate that the perturbed ROL-6::wrmScarlet distribution and localization exhibited in *sma-3* and *sma-9* mutants and *dpy-11* RNAi was caused by disturbances in ER-specific processes, we targeted the ER chemically and genetically. We observed similar ROL-6::wrmScarlet subcellular distribution in tunicamycin treated ROL-6::wrmScarlet expressing animals as seen in *sma-3* and *sma-9* mutants (Figure 7m; Figure 7 – figure supplement 1). Tunicamycin inhibits the glycan biosynthesis pathway, disrupts ER-mediated secretion, and induces ER stress. Moreover, RNAi-mediated depletion of genes involved in various steps of ER-derived vesicle production and transport, including *C54H2.5* (SURF4 ortholog/ER cargo release), *F41C3.4* (GOLT1A/b ortholog/ER to Golgi apparatus transport), *Y25C1A.5* (COPB-1/COPI coat complex subunit), and *Y113G7A.3* (COPII coat complex subunit) increased ROL-6::wrmScarlet hypodermal intracellular accumulation and left empty patches in the cuticle, similar to BMP signaling mutants (Figure 6g; Figure 6 – figure supplement 1). To test whether impaired secretion through the ER alone could compromise body size, we examined nematodes treated with tunicamycin and found that they showed a strong decrease in body size (Figure 7n). Taken together, our results suggest that signaling by DBL-1/BMP signaling promotes body size growth by promoting ER-specific processes involved in collagen maturation, transport, and secretion into the cuticular ECM.

## DISCUSSION

TGF-β signaling pathways regulate sets of target genes to execute biological functions in a context-dependent manner. Canonical signaling is mediated by the Smad transcription factor complex, but Smad binding sites are too degenerate and low affinity to account for the specific context-dependent effects, so transcription factor partners must also be involved. Some of the characterized partners are cell type-specific transcription factors. In contrast, Schnurri proteins are transcriptional partners that co-regulate target genes across multiple cell types. Here we used genome-wide RNA-seq and ChIP-seq and a novel software analysis pipeline to untangle the roles of Smad and Schnurri transcription factors in the developing *C. elegans* larva. We chose the second larval (L2) stage because Smad activity is elevated at this stage as determined by the RAD-SMAD activity reporter [57], and because this stage is the earliest point at which one can observe a clear difference for one of the best studied Smad mutant phenotypes: body size growth. Our analysis provides a complete picture of the direct targets of the DBL-1 signaling pathway during larval growth, and it identifies a novel set of effectors that mediate changes in body size in response to DBL-1 signaling, including factors involved in collagen secretion into the cuticle.

By using an analysis pipeline that combines BETA, which integrates ChIP-seq and RNA-seq data to identify targets, with LOA, which integrates two separate RNA-seq pairwise comparisons to identify shared DEGs, we demonstrated that SMA-3/Smad acts primarily as a transcriptional activator, whereas SMA-9/Schnurri can function as either an activator or a repressor depending on the locus. These dual functions for SMA-9/Schnurri are consistent with previous studies that demonstrated that different domains of SMA-9 can act as activators or repressors in a heterologous system [34]. Furthermore, SMA-9 DNA-binding domain fusions with known transcriptional activation or repression domains could each rescue a subset of mutant defects in *sma-9* mutants [34].

Our analysis revealed that SMA-3/Smad and SMA-9/Schnurri have both co-regulated and independently regulated target genes. Interestingly, the GO terms for both shared and independent target genes partially overlap, suggesting broad similarity in biological functions. In vertebrates, Schnurri homologs are shown to be direct DNA binding proteins with diverse biological functions that include TGF-β-responsive and TGF-β-independent roles. In TGF-β-independent roles they bind NFκB-like sequences [62], and can interact with other transcription factors including TRAF2 and c-Jun [63, 64]. It will be interesting to determine whether the SMA-9/Schnurri-specific target genes are responsive to TGF-β signals and/or to other signaling ligands. Within the subset of shared target genes, our analysis indicated that a significant number have overlapping binding peaks, consistent with a model in which SMA-3 and SMA-9 bind as a complex. Complex formation is consistent with the model for Smad-Schnurri interaction in Drosophila and Xenopus. We have now generated new insights into how a Smad-Schnurri complex may regulate target gene expression in a locus-specific manner. Analysis of *sma-3; sma-9* double mutants suggests that these factors may have antagonistic effects in addition to cooperative effects. Antagonism would be consistent with the observed interaction between SMA-9 and the BMP pathway in mesodermal lineage specification [36, 65]. For co-activated target genes, genetic analysis is consistent with the proteins acting together rather than additively, consistent with complex formation.

To identify a mechanism for antagonism between SMA-3 and SMA-9, we analyzed the co-regulated target gene, C54E4.5, which is downregulated in *sma-3* and upregulated in *sma-9* mutants. In the *sma-3; sma-9* double mutant, C54E4.5 is upregulated as it is in *sma-9* single mutants. Thus, SMA-3 activation of C54E4.5 is dependent on the presence of SMA-9. This dependence could occur if SMA-9 is needed to recruit SMA-3 to the DNA. This possibility would be a novel mode of interaction, because for the previously analyzed *brinker* and *Xvent2* target genes, Schnurri was shown to be recruited by the Smad complex rather than vice versa. A second possibility is that SMA-3 can bind in the absence of SMA-9 but cannot engage with the transcriptional machinery; that is, both proteins are required to form a transcriptional activation complex. With either possibility, SMA-9 represses C54E4.5 expression in the absence of SMA-3.

Finally, GO term analysis readily identified target genes involved in lipid metabolism and pathogen response, but target genes required for body size regulation remain more difficult to predict. To circumvent this barrier, we performed body size measurements on mutants. We also performed body size measurements on RNAi knockdowns for identified target genes in an RNAi sensitive strain, examining their effect in both a wild-type background and a *lon-2* background in which DBL-1 signaling is exaggerated, resulting in an elongated body size. These analyses confirmed previous work focusing on the role of the cuticle in mediating body size regulation by DBL-1/BMP [35]. Although RNAi knockdowns of *clec-1, fah-1,* C52D10.3, *dre-1, hsp-12.3, wrt-1* reduced body size in the *rrf-3* mutant background, they failed to reduce the body size in *lon-2*; *rrf-3* mutants, suggesting that they regulate body size upstream or independently of the DBL-1 pathway. By contrast, RNAi knockdowns of *haf-9*, *his-32*, *zip-10*, *emb-8*, *F25B5.6*, *nath-10*, and *hsp-3* reduced body size in *lon-2; rrf-3* double mutants but not in *rrf-3* single mutants, suggesting that they might be factors whose effects are only detectable in the context of an overactive pathway; these warrant future study. Seventeen genes showed a body reduction with a statistically significant effect size equal to or greater than one in both genetic backgrounds, making them of particular interest. These genes had GO terms associated with either one-carbon metabolism or chaperone/ER secretion, suggesting that the upregulation of these activities is a key aspect of how DBL-1 signaling promotes growth.

How could one-carbon metabolism play a role in body size growth? This complex set of interlinked metabolic cycles is critical for methionine and folate homeostasis. It also provides the methyl groups needed to synthesize nucleotides, amino acids, the antioxidant glutathione, creatine, and phospholipids like phosphatidylcholine, a fundamental component of membranes [66]. One-carbon metabolism also provides the methyl groups needed to make epigenetic marks on DNA and chromatin. The specific role of this metabolic pathway in body size growth will be an important topic of future study.

An enrichment of ER secretion and chaperone factors in the list of direct targets involved in body size growth was unexpected, but reasonable given that one of the key functions of the hypodermis is to secrete cuticular collagen. The role of collagens in body size and morphology is well documented [29, 67], and the secreted ADAMTS metalloprotease ADT-2 modifies cuticle collagen organization and regulates body size [68]. To test whether DBL-1 regulates ER secretion, we turned to endogenously tagged cuticle collagen ROL-6::wrmScarlet to analyze subcellular localization. Using this reporter for collagen synthesis and secretion, we demonstrated that the DBL-1 pathway influences the secretion of this cuticle collagen. In particular, ROL-6::wrmScarlet accumulates in a perinuclear ER compartment in DBL-1 pathway mutants. Interestingly, treatment with tunicamycin, which impairs ER secretion, was sufficient to reduce body size, which is consistent with a model in which BMP signaling promotes collagen secretion to foster growth. We remain cautious in our interpretation of this result, as blocking ER secretion with tunicamycin could affect the secretion of the BMP receptors or other proteins that function together with the receptors, which could also lead to a body size defect.

The collagenous cuticle is a major target of DBL-1/BMP signaling in body size regulation. For example, others have also demonstrated that DBL-1 signaling regulates cuticle collagen LON-3 post-transcriptionally [69]. In addition to direct transcriptional regulation of cuticle components, here we highlight the importance of regulating other target genes such as *dpy-11* that are needed for collagen post-transcriptional processing. TGF-β family members are well known regulators of collagen deposition and extracellular matrix composition, suggesting that this class of transcriptional targets is conserved over evolutionary time [70, 71]. Thus, it is likely that the multi-level interactions identified in *C. elegans* are also relevant to the functions of these factors in vertebrates.

## Supplementary Material

Supplement 1 Figure 3 – figure supplement 1. DAVID annotation clusters of gene ontology terms for identified SMA-3 and SMA-9 targets.
Tables of annotation clusters for gene ontology terms for direct target DEGs (via DAVID, with accompanying statistical EASE score) are shown for (a) SMA-3 and (b) SMA-9.
Figure 5 – figure supplement 1. Target genes required for exaggerated growth in *lon-2* mutants
(a) Mean body length of L4 animals (normalized to EV) for the indicated RNAi knockdowns in the *rrf-3; lon-2* double mutant. “EV” indicates the empty RNAi vector as a negative control. Dots indicate the size of individual animals. Asterisks above each column indicate one way ANOVA with Dunnett’s multiple comparison test against EV (****P<0.0001, ***P<0.001, **P<0.01, *P<0.05). The dotted line indicates the size value falling two standard deviations below the mean of EV (b) Glass’ effect size (the difference between the mean of the RNAi knockdown and the mean for EV, divided by the standard deviation for EV) for the indicated gene undergoing knockdown. The dotted line indicates an effect size of one. Green bars indicate genotypes for which the RNAi knockdown resulted in a statistically significant reduction (P<0.05) with a Glass’ effect size of one or greater. (c) The average of the Glass’ effect size between the *rrf-3* wild-type background and the *rrf-3 lon-2* mutant background (individual dots for each are shown). For (c), yellow bars indicate genotypes for which the RNAi knockdown resulted in a statistically significant reduction (P<0.05) with a Glass’ effect size of one or greater in both the wild-type and the *lon-2* mutant background. For all graphs, red and blue columns indicate values for *sma-3* and *sma-9* RNAi knockdown, respectively.
Figure 6 – figure supplement 1. DPY-11 promotes body size through collagen secretion
(a) ROL-6::wrmScarlet fluorescence detected in annuli and alae in the cuticular layer, as well as in nuclear envelopes in the underlying hypodermal layer in wild-type animals exposed to an empty vector for RNAi knockdown. (b-f) ROL-6::wrmScarlet in animals exposed to RNAi knockdown for the indicated gene. (b) In animals knocked down for *dpy-11*, little ROL-6::wrmScarlet makes it to the cuticle, instead accumulating intracellularly in the hypodermis. (c-f) In animals knocked down for known ER secretory factors, patches of cuticular surface show diminished levels of ROL-6::wrmScarlet, whereas the protein is detected in the hypodermal layer just under these patches (yellow arrowheads), similar to what is observed in *sma-3* and *sma-9* mutants, and consistent with a failure to deliver collagen to the surface cuticle. The bar indicates 5 microns.
Figure 7 – figure supplement 1. Tunicamycin treatment mimics the effect of *sma-3* and *sma-9* mutations on collagen secretion
(a) ROL-6::wrmScarlet fluorescence detected in annuli and alae in the cuticular layer, as well as in nuclear envelopes in the underlying hypodermal layer in untreated wild-type animals. (b) ROL-6::wrmScarlet in animals exposed to tunicamycin. Patches of cuticular surface show diminished levels of ROL-6::wrmScarlet, whereas the protein is detected in the hypodermal layer just under these patches (yellow arrowheads), similar to what is observed in *sma-3* and *sma-9* mutants, and consistent with a failure to deliver collagen to the surface cuticle. In addition, collagen in the lateral alae is disorganized, suggesting secretion is impaired in the seam cells. The bar indicates 5 microns.
