# Supplementary material for: Genome-wide analysis of Smad and Schnurri transcription factors in C. elegans demonstrates widespread interaction and a function in collagen secretion
Source: bioRxiv. 2024 Nov 14:2024.06.05.597576. Originally published 2024 Jun 6. Preprint. [Version 2] doi: 10.1101/2024.06.05.597576 (PMC11185707; doi:10.1101/2024.06.05.597576)

bioRxiv preprint doi: https://doi.org/10.1101/2024.06.05.597576; this version posted November 14, 2024. The copyright holder for this preprint (which was not certified by peer review) is the author/funder, who has granted bioRxiv a license to display the preprint in perpetuity. It is made available under aCC-BY 4.0 International license.

Figure 3 – figure supplement 1. DAVID annotation clusters of gene ontology terms for identified SMA-3 and SMA-9 targets.

Tables of annotation clusters for gene ontology terms for direct target DEGs (via DAVID, with accompanying statistical EASE score) are shown for (a) SMA-3 and (b) SMA-9.

Figure 3 – figure supplement 2. SMA-3 and SMA-9 sites tend not to be at HOT sites.

(a,b) Cumulative probability distributions measuring the number of (a) SMA-3 or (b) SMA-9 ChIP-seq peaks against the number of other transcription factors known from ModENCODE/ModERN analysis to bind to each site's region of the genome (within 400 bps of each ChIP-seq peak center). The black line indicates all SMA-3 or SMA-9 ChIP-seq peaks, whereas the magenta line indicates functional SMA-3 or SMA-9 ChIP-seq peaks identified by BETA analysis. The cyan line indicates SMA-3 and SMA-9 sites that overlap near co-regulated genes. The orange line indicates SMA-3-exclusive or SMA-9-exclusive sites. The red dotted vertical line with the rightward arrow indicates the minimum cutoff for high occupancy target (HOT) sites, which bind to 15 or more different transcription factors as determined by modENCODE/modERN. The black dotted horizontal line indicates the cumulative probability distribution percentage at which the curve for all ChIP-seq peaks intersects the cutoff for HOT sites (i.e., 15 or more TFs binding within 400 bps).

bioRxiv preprint doi: https://doi.org/10.1101/2024.06.05.597576; this version posted November 14, 2024. The copyright holder for this preprint (which was not certified by peer review) is the author/funder, who has granted bioRxiv a license to display the preprint in perpetuity. It is made available under aCC-BY 4.0 International license.

normalized to actin mRNA levels in that genotype. *P<0.05 Two-way ANOVA with Tukey's multiple comparison test. Data was analyzed from three biological replicates.

Figure 5 – figure supplement 1. Target genes required for exaggerated growth

(a) Mean body length of L4 animals (normalized to wild type) for the indicated mutants. Dots indicate the size of individual animals. Asterisks above each column indicate one way ANOVA with Dunnett's multiple comparison test against wild type (****P<0.0001, ***P<0.001, **P<0.01, *P<0.05). Asterisks over pairwise comparison bars indicate one-way ANOVA with Sídák's multiple comparison test. The dotted line indicates the size value falling two standard deviations below the mean of wild type. (b) Glass' effect size (the difference between the mean of the

bioRxiv preprint doi: https://doi.org/10.1101/2024.06.05.597576; this version posted November 14, 2024. The copyright holder for this preprint (which was not certified by peer review) is the author/funder, who has granted bioRxiv a license to display the preprint in perpetuity. It is made available under aCC-BY 4.0 International license.

mutant and the mean for wild type, divided by the standard deviation of wild type) for the indicated mutants. The dotted line indicates an effect size of one. (c,d) Mean body size and Glass' effect size for RNAi knockdowns of the indicated gene in the *rrf-3* RNAi sensitive strain. "EV" indicates the empty RNAi vector as a negative control. Asterisks above each column indicate one way ANOVA with Dunnett's multiple comparison test against EV (****P<0.0001, ***P<0.001, **P<0.01, *P<0.05). For all graphs, red, blue, and purple columns indicate values for *sma-3* (mutant or RNAi knockdown), *sma-9* (mutant or RNAi knockdown), and their double mutant combination, respectively. Green bars indicate genotypes for which the mutant or RNAi knockdown resulted in a statistically significant reduction (P<0.05) with a Glass' effect size of one or greater.

Figure 5 – figure supplement 2. Target genes required for exaggerated growth in *lon-2* mutants

(a) Mean body length of L4 animals (normalized to EV) for the indicated RNAi knockdowns in the *rrf-3; lon-2* double mutant. "EV" indicates the empty RNAi vector as a negative control. Dots indicate the size of individual animals. Asterisks above each column indicate one way ANOVA with Dunnett's multiple comparison test against EV (****P<0.0001, ***P<0.001, **P<0.01, *P<0.05). The dotted line indicates the size value falling two standard deviations below the mean of EV (b) Glass' effect size (the difference between the mean of the RNAi knockdown and the mean for EV, divided by the standard deviation for EV) for the indicated gene undergoing knockdown. The dotted line indicates an effect size of one. Green bars indicate genotypes for which the RNAi knockdown resulted in a statistically significant reduction (P<0.05) with a Glass' effect size of one or greater. (c) The average of the Glass' effect size between the *rrf-3* wild-type background and the *rrf-3 lon-2* mutant background (individual dots for each are shown). For (c), yellow bars indicate genotypes for which the RNAi knockdown resulted in a statistically significant reduction (P<0.05) with a Glass' effect size of one or greater in both the wild-type and

bioRxiv preprint doi: https://doi.org/10.1101/2024.06.05.597576; this version posted November 14, 2024. The copyright holder for this preprint (which was not certified by peer review) is the author/funder, who has granted bioRxiv a license to display the preprint in perpetuity. It is made available under aCC-BY 4.0 International license.

the *lon-2* mutant background. For all graphs, red and blue columns indicate values for *sma-3* and *sma-9* RNAi knockdown, respectively.

bioRxiv preprint doi: https://doi.org/10.1101/2024.06.05.597576; this version posted November 14, 2024. The copyright holder for this preprint (which was not certified by peer review) is the author/funder, who has granted bioRxiv a license to display the preprint in perpetuity. It is made available under aCC-BY 4.0 International license.

Figure 6 – figure supplement 1. DPY-11 promotes body size through collagen secretion

(a) ROL-6::wrmScarlet fluorescence detected in annuli and alae in the cuticular layer, as well as in nuclear envelopes in the underlying hypodermal layer in wild-type animals exposed to an empty vector for RNAi knockdown. (b-f) ROL-6::wrmScarlet in animals exposed to RNAi knockdown for the indicated gene. (b) In animals knocked down for *dpy-11*, little ROL-6::wrmScarlet makes it to the cuticle, instead accumulating intracellularly in the hypodermis. (c-f) In animals knocked down for known ER secretory factors, patches of cuticular surface show diminished levels of ROL-6::wrmScarlet, whereas the protein is detected in the hypodermal layer just under these patches (yellow arrowheads), similar to what is observed in *sma-3* and *sma-9* mutants, and consistent with a failure to deliver collagen to the surface cuticle. The bar indicates 5 microns.

bioRxiv preprint doi: https://doi.org/10.1101/2024.06.05.597576; this version posted November 14, 2024. The copyright holder for this preprint (which was not certified by peer review) is the author/funder, who has granted bioRxiv a license to display the preprint in perpetuity. It is made available under aCC-BY 4.0 International license.

Figure 7 – figure supplement 1. Tunicamycin treatment mimics the effect of *sma-3* and *sma-9* mutations on collagen secretion

(a) ROL-6::wrmScarlet fluorescence detected in annuli and alae in the cuticular layer, as well as in nuclear envelopes in the underlying hypodermal layer in untreated wild-type animals. (b) ROL-6::wrmScarlet in animals exposed to tunicamycin. Patches of cuticular surface show diminished levels of ROL-6::wrmScarlet, whereas the protein is detected in the hypodermal layer just under these patches (yellow arrowheads), similar to what is observed in *sma-3* and *sma-9* mutants, and consistent with a failure to deliver collagen to the surface cuticle. In addition, collagen in the lateral alae is disorganized, suggesting secretion is impaired in the seam cells. The bar indicates 5 microns.

bioRxiv preprint doi: https://doi.org/10.1101/2024.06.05.597576; this version posted November 14, 2024. The copyright holder for this preprint (which was not certified by peer review) is the author/funder, who has granted bioRxiv a license to display the preprint in perpetuity. It is made available under aCC-BY 4.0 International license.

SUPPLEMENTAL FILES

Supplemental File 1. SMA-3 and SMA-9 ChIP-seq Sites.

This file contains the chromosomal location of 4205 ChIP-seq peaks for SMA-3 and 7065 ChIP-seq peaks for SMA-9 in separate tabs labeled "SMA-3" and "SMA-9," respectively. SMA-3 sites that overlap with a SMA-9 site are listed on the "Overlapping Sites_S3" tab. SMA-9 sites that overlap with a SMA-3 site are listed on the "Overlapping Sites_S9" tab. Non-overlapping SMA-3 and SMA-9 sites are listed on the "Non-overlapping_S3" and "Non-overlapping_S9" tabs, respectively. For all tabs, column A indicates chromosome location, column B indicates the start of the peak sequence, and column C indicates the end of the peak sequence. Column labels are in row 1.

Supplemental File 2. Differential Gene Expression From *sma-3* and *sma-9* Mutants.

This file lists the differential gene expression from RNA-seq of *sma-3* vs wild type (the tab labeled "SMA3 vs N2"), as well as *sma-9* versus wild type (the tab labeled "SMA9 vs N2"). For each gene, WormBase GeneID, public gene name, log fold change, p-value, and FDR are listed. Column labels are in row 1.

Supplemental File 3. SMA-3 and SMA-9 Direct Targets.

This file lists the direct target genes identified by BETA analysis of RNA-seq and ChIP-seq data. Direct targets of SMA-3 are in the tab labeled "SMA3 Direct Targets." Direct targets of SMA-9 are in the tab labeled "SMA9 Direct Targets." For each gene, chromosomal location, transcriptional start site, transcriptional end site, public gene name, rank product from BETA analysis, RNA-seq log fold change from corresponding mutant vs wild type, and WormBase GeneID are listed. Column labels are in row 1.

bioRxiv preprint doi: https://doi.org/10.1101/2024.06.05.597576; this version posted November 14, 2024. The copyright holder for this preprint (which was not certified by peer review) is the author/funder, who has granted bioRxiv a license to display the preprint in perpetuity. It is made available under aCC-BY 4.0 International license.

Supplemental File 4. Differential Gene Expression Shared Between *sma-3* and *sma-9* Mutants Analyzed Using LOA.

This file lists the differentially expressed genes identified by LOA analysis as being common to both the RNA-seq of *sma-3* vs wild type as well as the RNA-seq of *sma-9* versus wild type. For each gene, WormBase GeneID and public gene name are listed, followed by the log fold change, p-value, and FDR from the *sma-3* vs wild type RNA-seq, followed by the log fold change, p-value, and FDR from the *sma-9* vs wild type RNA-seq. Column labels are in row 1.

Supplemental File 5. Classes of Direct Targets for SMA-3 and SMA-9.

This file lists the direct target genes identified by combined LOA/BETA analysis of RNA-seq and ChIP-seq data, as described in Figure 3. Direct targets of SMA-3 alone are in the tab labeled "Figure 3b." Direct targets of SMA-3 and SMA-9 in which both factors promote the target's expression are in the tab labeled "Figure 3c." Direct targets of SMA-3 and SMA-9 in which the two factors have opposite effects on the target's expression are in the tab labeled "Figure 3d." Direct targets of SMA-9 alone in which the factor either promotes or inhibits the target's expression are in the tabs labeled "Figure 3e" and "Figure 3f," respectively. For each gene, WormBase GeneID and public gene name are listed, followed by the log fold change and FDR from the *sma-3* vs wild type RNA-seq, followed by the log fold change and FDR from the *sma-9* vs wild type RNA-seq. Column labels are in row 1.

bioRxiv preprint doi: https://doi.org/10.1101/2024.06.05.597576; this version posted November 14, 2024. The copyright holder for this preprint (which was not certified by peer review) is the author/funder, who has granted bioRxiv a license to display the preprint in perpetuity. It is made available under aCC-BY 4.0 International license.

Figure 3 – figure supplement 1

a

SMA-3 Direct Targets Annotation Clusters

Annotation Cluster	EASE Score
Ribosome Biogenesis	6.18
Oxido-reductases	3.31
Mitochondria	3.10
One-carbon Metabolism	3.03
RNA Helicases	2.95
ER Chaperones	2.89
Chromosome Organization	2.66
mRNA Splicing	2.64
Fatty Acid Metabolism	2.58
Amino Acid Biosynthesis	2.25
RNA Binding	2.08
Methionine Biosynthesis	2.00
Apple Domain	1.86
Acyl-CoA Dehydrogen	1.74
Isomerases	1.67
DNAJ Domain	1.46

b

SMA-9 Direct Targets Annotation Clusters

Annotation Cluster	EASE Score
Innate Immunity	7.44
Fatty Acid Metabolism	4.03
One-carbon Metabolism	3.23
Amino Acid Metabolism	2.73
Anti-Bacterial	2.67
Fatty Acid Transporters	2.62
Cytochrome P450	2.58
Lysosomes & Proteolysis	2.56
Cofactor Biosynthesis	2.33
Crontonases	2.14
Long FA Metabolism	2.05
Glutathione Metabolism	1.61
C-type Lectins	1.60

bioRxiv preprint doi: https://doi.org/10.1101/2024.06.05.597576; this version posted November 14, 2024. The copyright holder for this preprint (which was not certified by peer review) is the author/funder, who has granted bioRxiv a license to display the preprint in perpetuity. It is made available under aCC-BY 4.0 International license.

Figure 3 – figure supplement 2

a

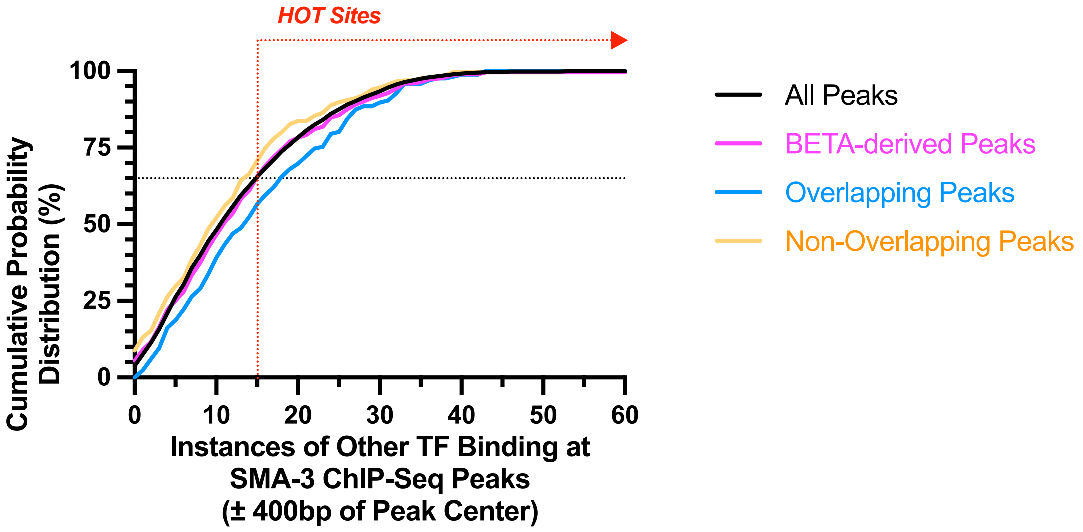

b

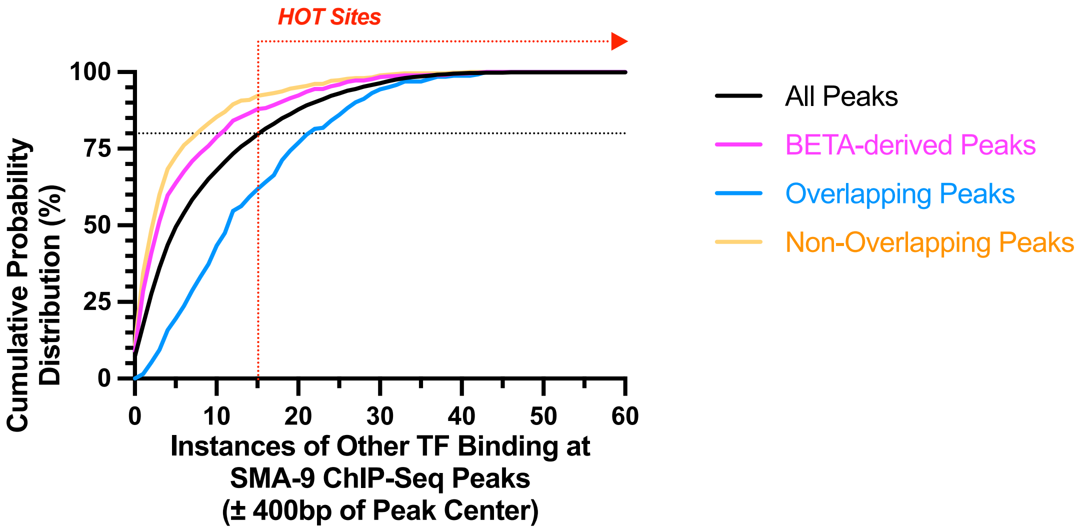

bioRxiv preprint doi: https://doi.org/10.1101/2024.06.05.597576; this version posted November 14, 2024. The copyright holder for this preprint (which was not certified by peer review) is the author/funder, who has granted bioRxiv a license to display the preprint in perpetuity. It is made available under aCC-BY 4.0 International license.

Figure 4 – supplement 1

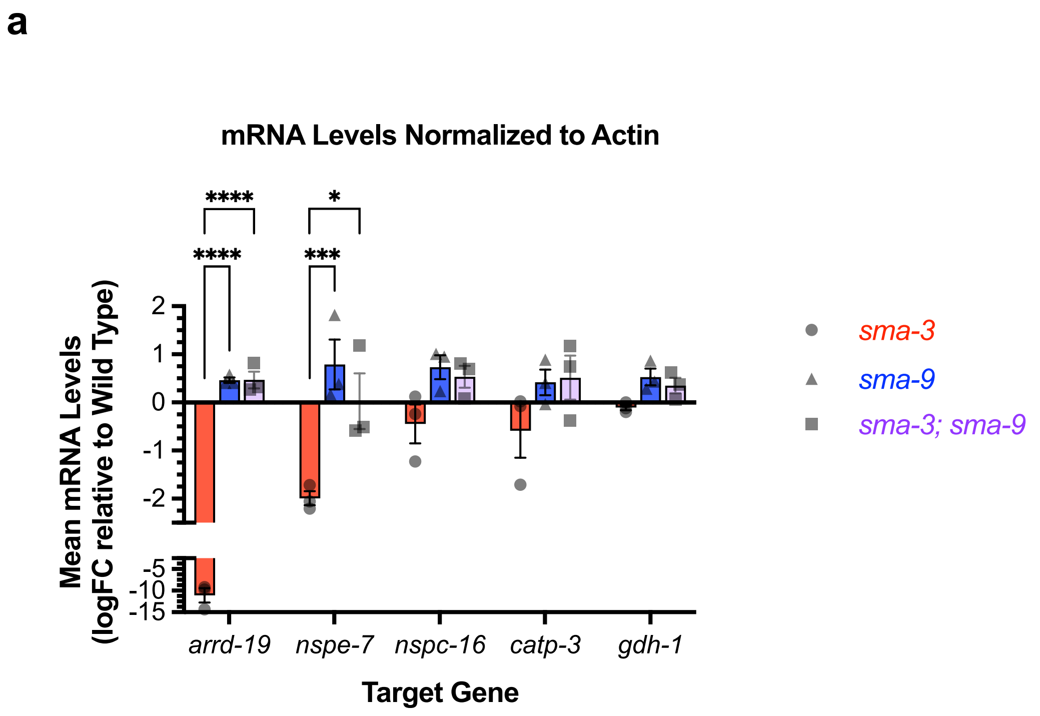

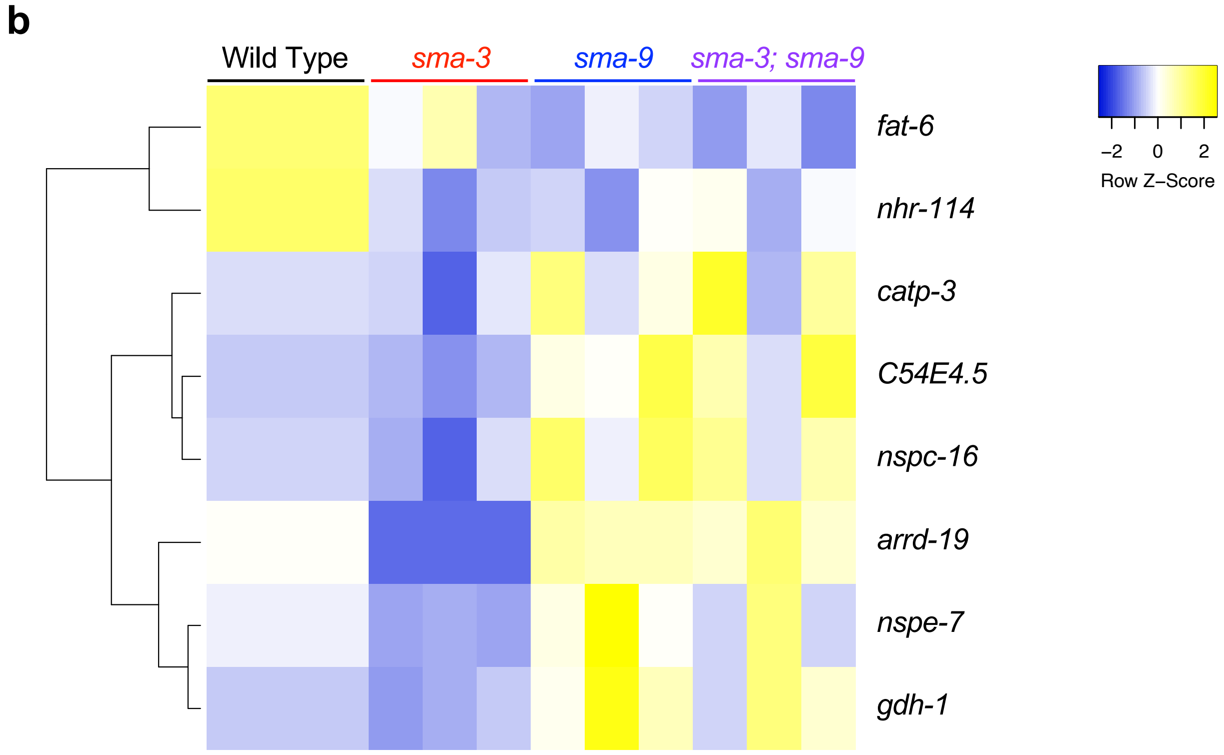

bioRxiv preprint doi: https://doi.org/10.1101/2024.06.05.597576; this version posted November 14, 2024. The copyright holder for this preprint (which was not certified by peer review) is the author/funder, who has granted bioRxiv a license to display the preprint in perpetuity. It is made available under aCC-BY 4.0 International license.

Figure 5 Supplement 1

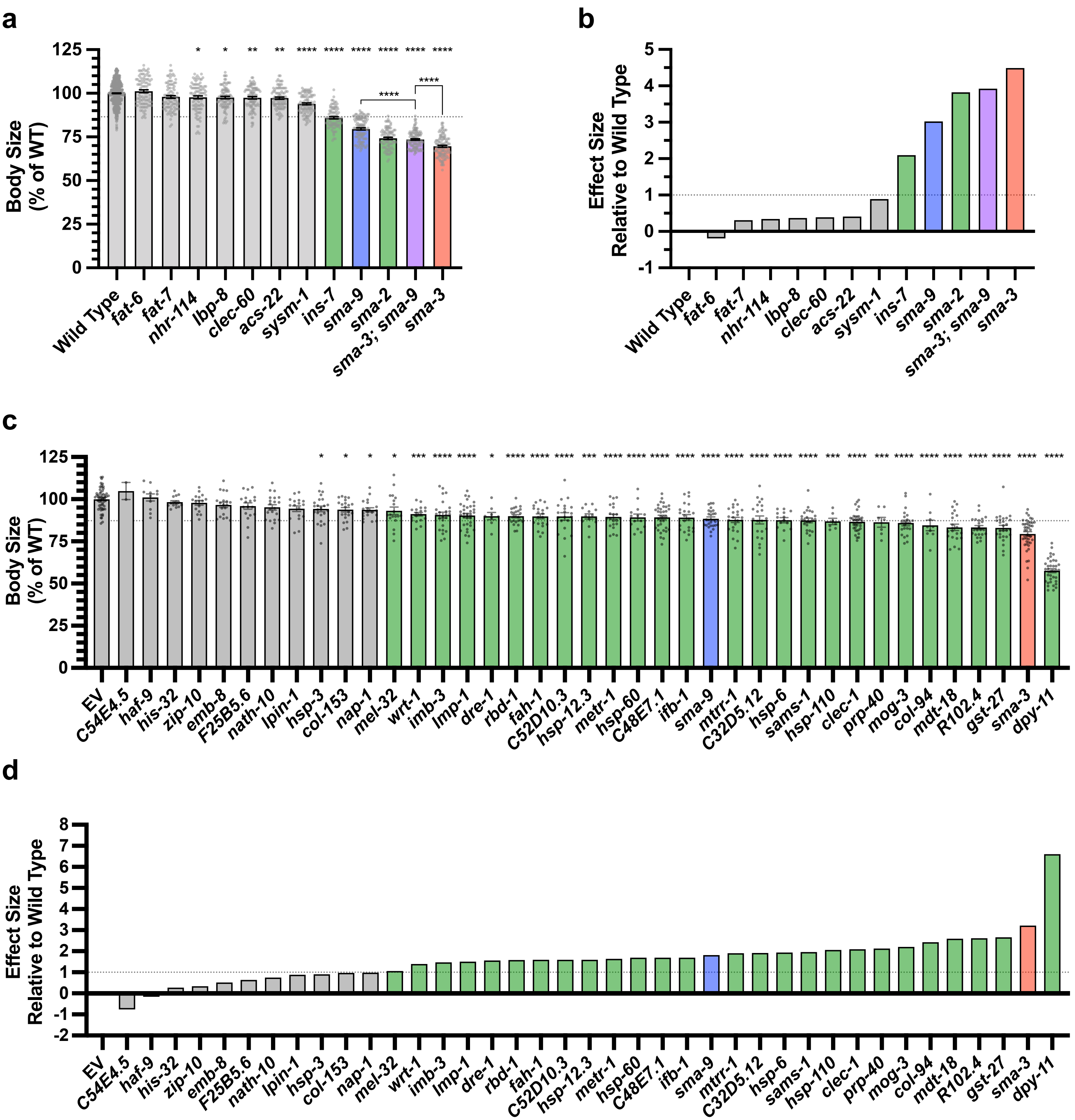

bioRxiv preprint doi: https://doi.org/10.1101/2024.06.05.597576; this version posted November 14, 2024. The copyright holder for this preprint (which was not certified by peer review) is the author/funder, who has granted bioRxiv a license to display the preprint in perpetuity. It is made available under aCC-BY 4.0 International license.

Figure 5 – figure supplement 2

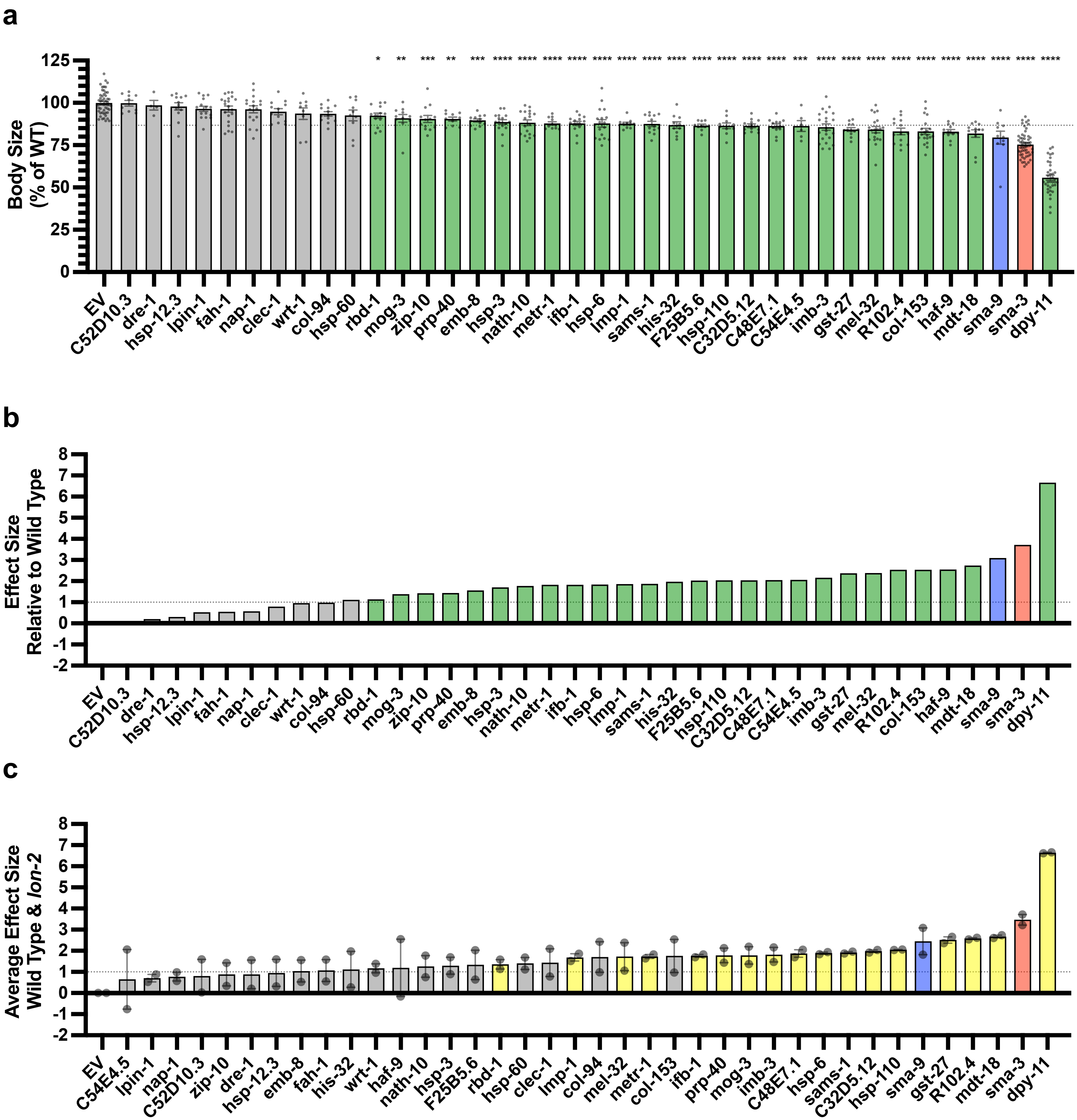

bioRxiv preprint doi: https://doi.org/10.1101/2024.06.05.597576; this version posted November 14, 2024. The copyright holder for this preprint (which was not certified by peer review) is the author/funder, who has granted bioRxiv a license to display the preprint in perpetuity. It is made available under aCC-BY 4.0 International license.

Figure 6 – figure supplement 1

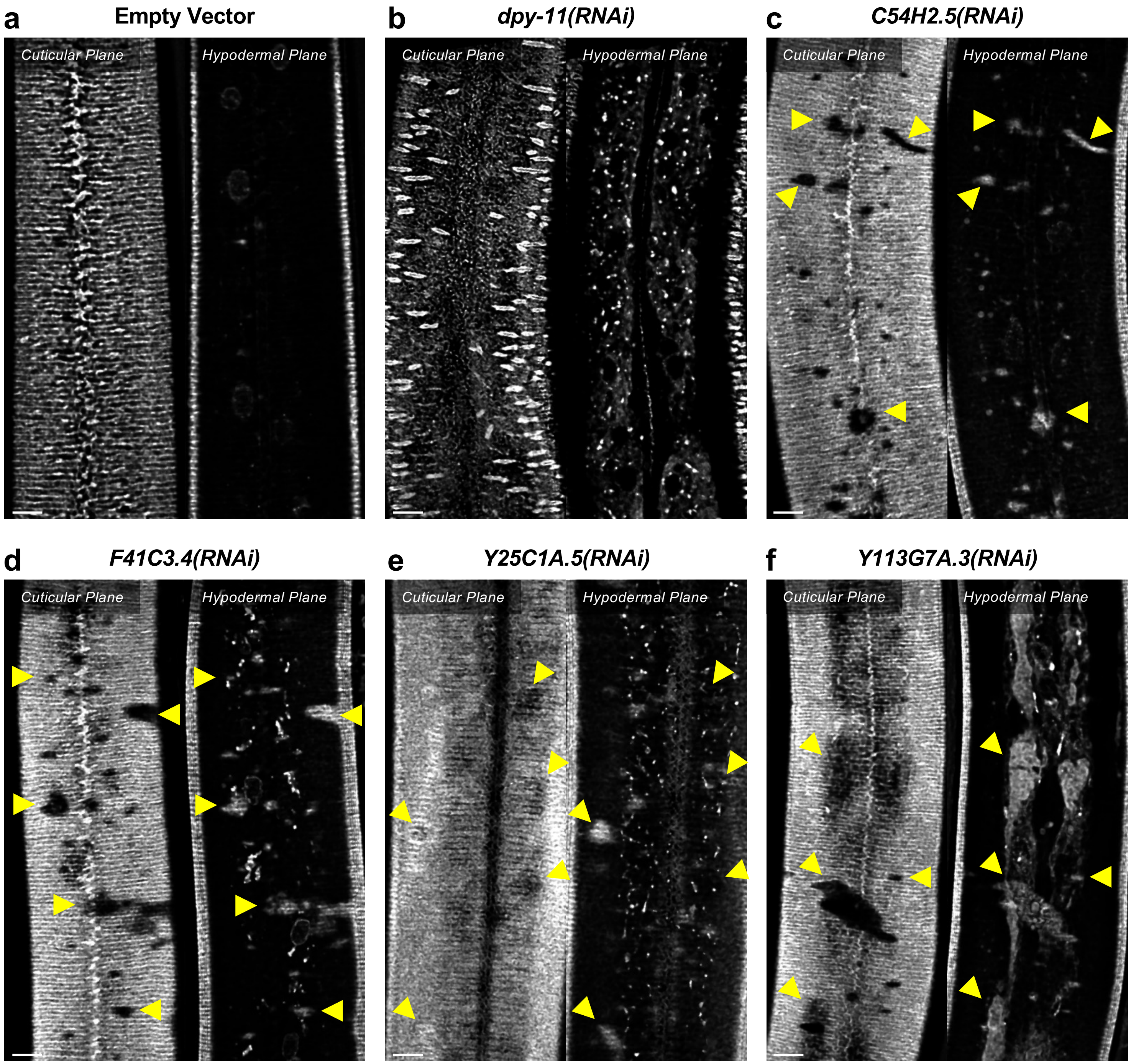

bioRxiv preprint doi: https://doi.org/10.1101/2024.06.05.597576; this version posted November 14, 2024. The copyright holder for this preprint (which was not certified by peer review) is the author/funder, who has granted bioRxiv a license to display the preprint in perpetuity. It is made available under aCC-BY 4.0 International license.

Figure 7 – figure supplement 1

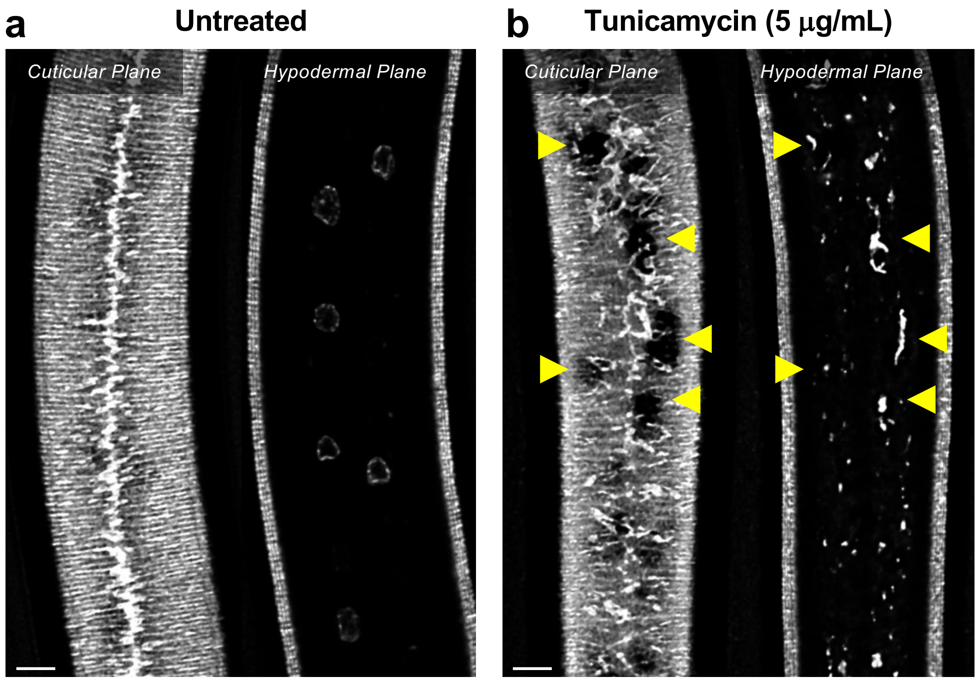

Supplement: Supplementary file 1 [file NIHPP2024.06.05.597576v2-supplement-6.pdf]
