# Supplementary material for: Genome-wide analysis of Smad and Schnurri transcription factors in C. elegans demonstrates widespread interaction and a function in collagen secretion
Source: bioRxiv. 2024 Jun 6:2024.06.05.597576. Preprint. [Version 1] doi: 10.1101/2024.06.05.597576 (PMC11185707; doi:10.1101/2024.06.05.597576)

bioRxiv preprint doi: https://doi.org/10.1101/2024.06.05.597576; this version posted June 6, 2024. The copyright holder for this preprint (which was not certified by peer review) is the author/funder, who has granted bioRxiv a license to display the preprint in perpetuity. It is made available under aCC-BY 4.0 International license.

Figure 3 – figure supplement 1

a

SMA-3 Direct Targets Annotation Clusters

Annotation Cluster	EASE Score
Ribosome Biogenesis	6.18
Oxido-reductases	3.31
Mitochondria	3.10
One-carbon Metabolism	3.03
RNA Helicases	2.95
ER Chaperones	2.89
Chromosome Organization	2.66
mRNA Splicing	2.64
Fatty Acid Metabolism	2.58
Amino Acid Biosynthesis	2.25
RNA Binding	2.08
Methionine Biosynthesis	2.00
Apple Domain	1.86
Acyl-CoA Dehydrogen	1.74
Isomerases	1.67
DNAJ Domain	1.46

b

SMA-9 Direct Targets Annotation Clusters

Annotation Cluster	EASE Score
Innate Immunity	7.44
Fatty Acid Metabolism	4.03
One-carbon Metabolism	3.23
Amino Acid Metabolism	2.73
Anti-Bacterial	2.67
Fatty Acid Transporters	2.62
Cytochrome P450	2.58
Lysosomes & Proteolysis	2.56
Cofactor Biosynthesis	2.33
Crontonases	2.14
Long FA Metabolism	2.05
Glutathione Metabolism	1.61
C-type Lectins	1.60

bioRxiv preprint doi: https://doi.org/10.1101/2024.06.05.597576; this version posted June 6, 2024. The copyright holder for this preprint (which was not certified by peer review) is the author/funder, who has granted bioRxiv a license to display the preprint in perpetuity. It is made available under aCC-BY 4.0 International license.

Figure 5 – figure supplement 1

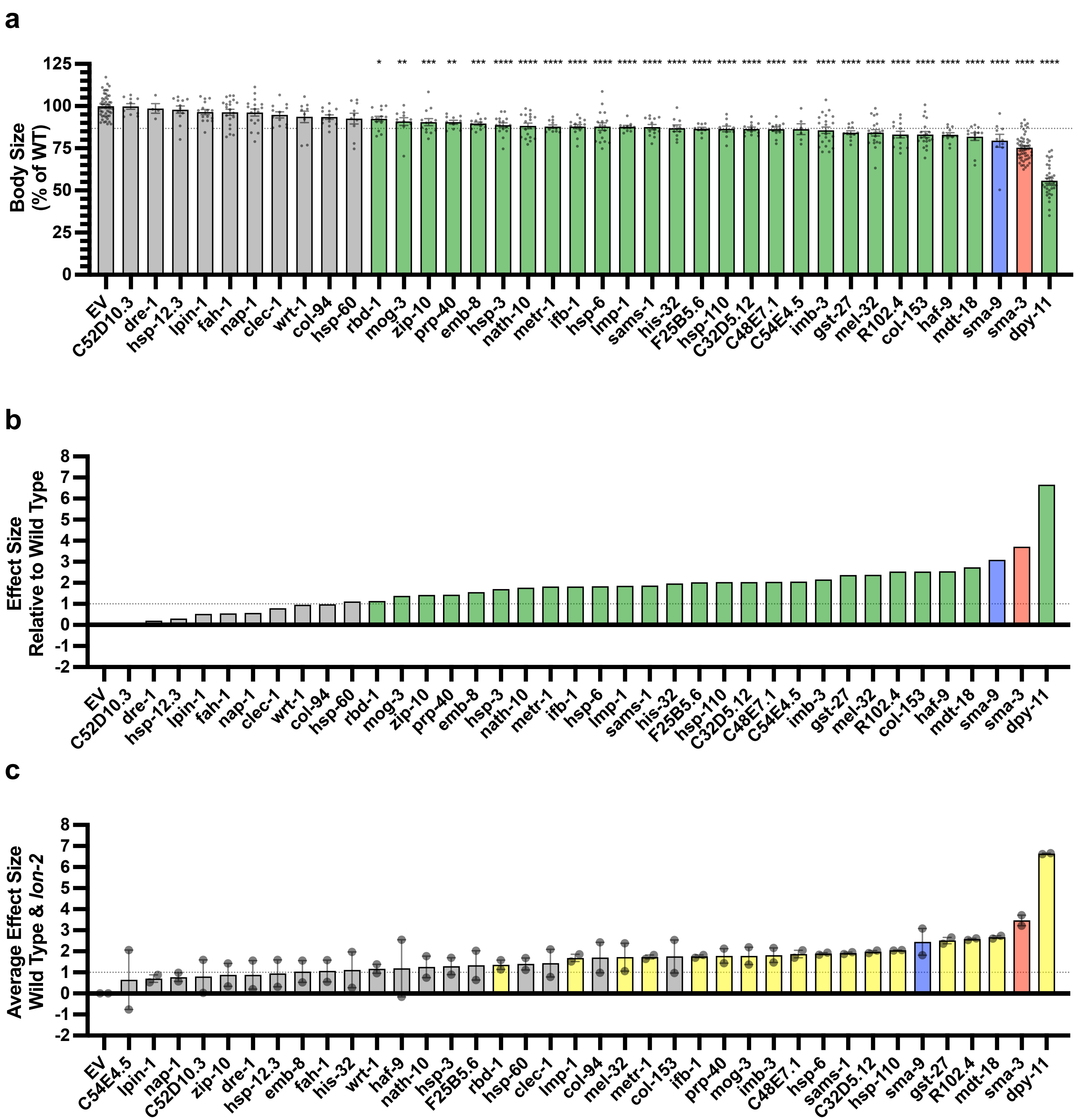

bioRxiv preprint doi: https://doi.org/10.1101/2024.06.05.597576; this version posted June 6, 2024. The copyright holder for this preprint (which was not certified by peer review) is the author/funder, who has granted bioRxiv a license to display the preprint in perpetuity. It is made available under aCC-BY 4.0 International license.

Figure 6 – figure supplement 1

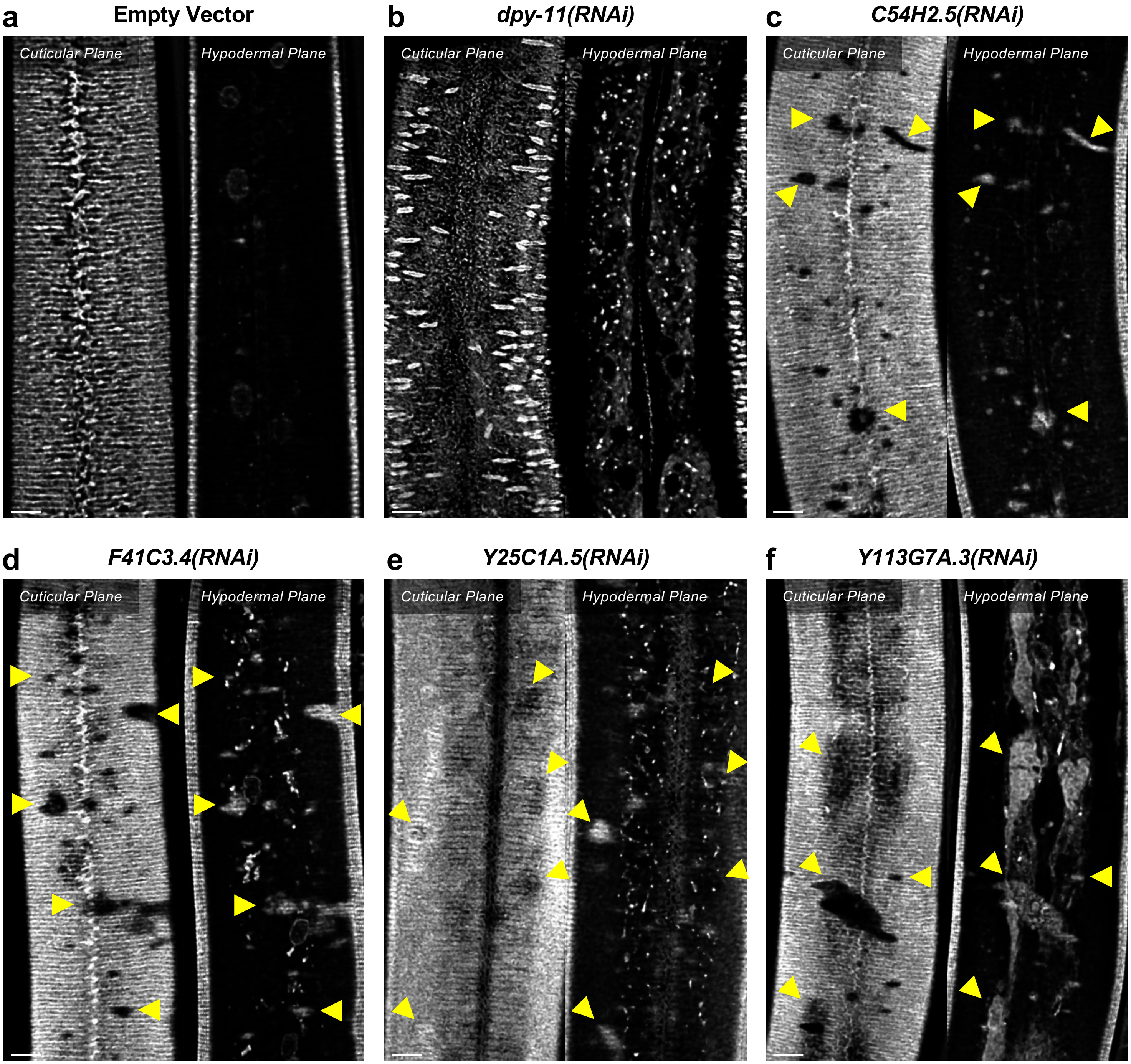

bioRxiv preprint doi: https://doi.org/10.1101/2024.06.05.597576; this version posted June 6, 2024. The copyright holder for this preprint (which was not certified by peer review) is the author/funder, who has granted bioRxiv a license to display the preprint in perpetuity. It is made available under aCC-BY 4.0 International license.

Figure 7 – figure supplement 1

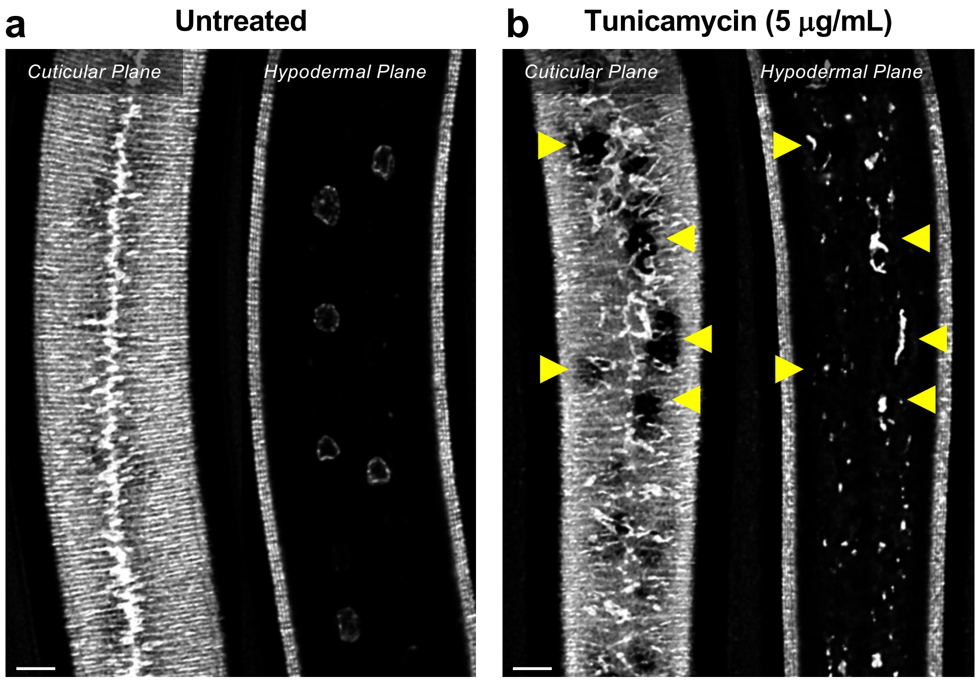

Supplement: Supplementary file 1 [file NIHPP2024.06.05.597576v1-supplement-1.pdf]
